# DOGS: Reaction-Driven *de novo* Design of Bioactive Compounds

**DOI:** 10.1371/journal.pcbi.1002380

**Published:** 2012-02-16

**Authors:** Markus Hartenfeller, Heiko Zettl, Miriam Walter, Matthias Rupp, Felix Reisen, Ewgenij Proschak, Sascha Weggen, Holger Stark, Gisbert Schneider

**Affiliations:** 1 Swiss Federal Institute of Technology (ETH), Department of Chemistry and Applied Biosciences, Institute of Pharmaceutical Sciences, Zürich, Switzerland; 2 Goethe-University, Institute of Pharmaceutical Chemistry, LiFF/OSF/ZAFES, Frankfurt am Main, Germany; 3 Department of Neuropathology, Heinrich-Heine-University, Düsseldorf, Germany; University of Houston, United States of America

## Abstract

We present a computational method for the reaction-based *de novo* design of drug-like molecules. The software DOGS (Design of Genuine Structures) features a ligand-based strategy for automated ‘in silico’ assembly of potentially novel bioactive compounds. The quality of the designed compounds is assessed by a graph kernel method measuring their similarity to known bioactive reference ligands in terms of structural and pharmacophoric features. We implemented a deterministic compound construction procedure that explicitly considers compound synthesizability, based on a compilation of 25'144 readily available synthetic building blocks and 58 established reaction principles. This enables the software to suggest a synthesis route for each designed compound. Two prospective case studies are presented together with details on the algorithm and its implementation. *De novo* designed ligand candidates for the human histamine H_4_ receptor and γ-secretase were synthesized as suggested by the software. The computational approach proved to be suitable for scaffold-hopping from known ligands to novel chemotypes, and for generating bioactive molecules with drug-like properties.

## Author Summary

The computer program DOGS aims at the automated generation of new bioactive compounds. Only a single known reference compound is required to have the computer come up with suggestions for potentially isofunctional molecules. A specific feature of the algorithm is its capability to propose a synthesis plan for each designed compound, based on a large set of readily available molecular building blocks and established reaction protocols. The *de novo* design software provides rapid access to tool compounds and starting points for the development of a lead candidate structure. The manuscript gives a detailed description of the algorithm. Theoretical analysis and prospective case studies demonstrate its ability to propose bioactive, plausible and chemically accessible compounds.

## Introduction

*De novo* design aims at generating new chemical entities with drug-like properties and desired biological activities in a directed fashion [1], [2]. This goal corresponds to the major task of the early drug discovery process and comprises a considerable fraction of the effort spent by pharmaceutical companies and academic groups in order to develop new treatments for diseases. *De novo* design is complementary to high-throughput screening in its approach to find innovative entry points for drug development [3]. Instead of searching for bioactive molecules in large collections of physically available screening compounds, *de novo* design ‘invents’ chemical structures from scratch by assembling molecular fragments. Computer-assisted approaches to *de novo* design automate this process by generating hypothetical candidate structures *in silico*. Although related areas of computer-aided drug development (*e.g.* virtual screening, quantitative structure-activity relationship modeling) have gained substantial attention in terms of publication numbers, *de novo* design has witnessed a constant evolution ever since the first computational methods have emerged in the late 1980s [2]. A number of reviews on this topic have been published recently, providing a comprehensive overview of the field [1]–[4].

Most of the approaches to *de novo* design attempt to mimic the work of a medicinal chemist: molecules are synthesized (virtually assembled from fragments), tested for their biological activity (computationally evaluated by a scoring function), and the insight gained serves as the basis for the next round of compound generation (optimization). *De novo* design methods differ in the way they search for, assemble, and score the generated molecules. For example, scoring can either be performed by computing some similarity index of candidate compounds and known reference ligands (*ligand-based* approach) or based on the three-dimensional (3D) structure of a ligand-binding cavity (*receptor-based* approach). Irrespective of the particular technique used, automated *de novo* design has always been confronted with the issue of synthetic accessibility [1], [5]. It may be argued that this is one of the main reasons why *de novo* design software has only rarely been subjected to practical evaluation [3]. An overview of successful *de novo* design studies is provided in a recent review article by Kutchukian and Shakhnovich [4].

Only a small fraction of all molecules amenable to virtual construction can in fact be synthesized in a reasonable time frame and with acceptable effort. *De novo* design programs tackle this issue by employing rules to guide the assembly process. Such rules attempt to reflect chemical knowledge and thereby avoid the formation of implausible or unstable structures. For example, some assembly approaches prevent connections between certain atom types, and finally the formation of unwanted substructures [6], [7]. Other strategies employ chemistry-driven retrosynthetic rules capturing general principles of reaction classes [2]. A prominent example of this kind of rule set is the RECAP [8] (retrosynthetic combinatorial analysis procedure), which is also used by some *de novo* design tools [9]–[12]. The software SYNOPSIS [13] follows a conceptually even more elaborate approach by connecting available molecular building blocks using a set of known chemical reactions. This enables the software to suggest reasonable synthesis pathways along with each final compound.

Here, we present a new approach to computer-assisted *de novo* design of ligand candidate structures, and describe its implementation in the software tool DOGS (Design Of Genuine Structures). DOGS represents a medicinal chemistry-inspired method for the *de novo* design of drug-like compounds, placing special emphasis on the synthesizability of the designed molecules. The software not only suggests new compounds, but also provides at least one motivated, hypothetical synthesis pathway per ligand candidate structure. The assembly process is based on available molecular building blocks and a set of established reaction principles. This strategy forces the program to follow construction pathways that represent direct blueprints of possible synthesis routes. The synthesis pathways generated and output by the software include vendor catalog identifiers of the building blocks and references to the underlying synthesis protocols.

DOGS grows new molecules in a deterministic and stepwise process: in each step, complete enumeration of a subspace of all possible solutions is performed. Following a greedy strategy, top-scoring intermediate products are submitted to subsequent growing steps. The quality of designed (intermediate) products is assessed by a ligand-based scoring scheme. Similarity to a reference ligand is computed by a graph kernel method. Two different graph representations of molecules (*molecular graph* and *reduced graph*) have been implemented to allow for different levels of abstraction from the two-dimensional molecular structure.

In a recently published work, we have successfully applied DOGS in a first prospective study to designing a selective inhibitor of human Polo-like kinase 1 (Plk1) in its inactive (DFG-out activation-loop) conformation [14]. One of the compounds suggested by DOGS was selected for synthesis based on a series of post-design analyses and human inspection. Following the proposed synthesis route, the compound was accessible and found to have the desired biological effect and selectivity profile *in vitro*. The Plk1 study focused on the practical use case and only provides a brief description of the method. Here, we disclose the algorithmic details and give a full description of the implementation. We present a theoretical evaluation of the software with respect to general properties of designed compounds, and show its ability to suggest well-motivated bioisosteric replacements. We also present two new prospective case studies: Three compounds designed by DOGS (two suggested as modulators of γ-secretase and one as an antagonist of human histamine H_4_ receptor) were selected for chemical synthesis and subsequently tested for *in vitro* bioactivity. In all cases, the proposed synthesis plan was readily pursuable as suggested by the software.

## Methods

### Library of Chemical Reactions

**Figure 1:**
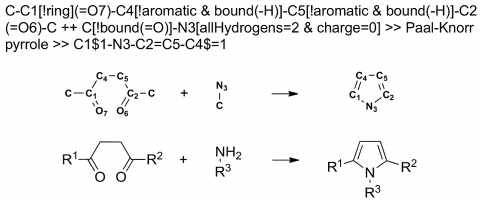
Encoding of reactions. Example of a Paal-Knorr pyrrole reaction encoded as Reaction-MQL expression (*top*). Reactant substructure descriptions (*left part*) are separated by ‘++’. The product (*right part*) is separated from the reactants by ‘>> *ID* >>’ where *ID* is an arbitrary identifier of the reaction. A direct structural representation of the line notation description including atom identifiers is shown in the center. The conventional structural representation of the reaction (*bottom*) denotes variable parts of molecules by *R*-groups (R^x^).

The DOGS algorithm builds up new candidate structures by mimicking a multi-step synthesis pathway. This strategy is supposed to deliver a direct blueprint for the actual synthesis of proposed candidate structures. For this approach, established reaction protocols need to be formalized in order to make them processable by a computer. Reactions were encoded using the formal language Reaction-MQL [15]. The specification of a reaction as a Reaction-MQL expression consists of a reactant side on the left and a product side on the right. A reactant is specified only by the substructure that is directly involved or essential for the reaction (*reaction center*) in order to make the description applicable to a broad spectrum of reactants with variable substituent groups (*R*-groups). The product is described by bond rearrangements caused by the reaction (Figure 1). All Reaction-MQL representations used in this work feature reactants with variable *R*-groups to keep them as generic as possible. Catalysts and invariant reactants are not denominated in the reaction expressions.

DOGS implements 83 reactions (termed *coupling reactions* in the following), 58 of which are unique and 25 represent either charge variations (reactants) or regioisomer variations (products) of one of the unique reactions. The complete list of reactions is provided in Table S1 in Text S1, supplementary material. Out of the 58 unique reactions, 34 describe ring formations. All reactions require one or two reactants (referred to as *one-* or *two-component reactions*, respectively) and result in a single product (A→B; A+B→C). In case a reaction generates regioisomers, it is split into two separate Reaction-MQL expressions, each describing one of the regioisomer products.

### Building Block Library for Virtual Synthesis

A subset of the Sigma-Aldrich (Sigma-Aldrich Co., 3050 Spruce St, St. Louis, MO 63103, USA) catalog containing 56,878 chemical building blocks was downloaded from the ZINC database [16], [17]. These compounds served as a basis for the extraction of the final set of building blocks by a three-step preparation protocol.

In the first step, building blocks were standardized, and unsuitable entries were eliminated. For this purpose, a preprocessing routine was developed and implemented using the software MOE (version 2009.10; Chemical Computing Group, Suite 910, 1010 Sherbrooke Street West, Montreal, Quebec, Canada):

- Compounds with a molecular mass of less than 30 Da or more than 300 Da were removed.
- Compounds containing more than four rings were removed.
- Compounds with any element type other than C, N, O, S, P, F, Cl, Br, I, B, Si, Se were removed.
- Compounds containing more than three fluoride atoms were removed.
- Compounds featuring atoms with incorrect valences were removed.
- Compounds containing unwanted substructures were removed according to the recommendations by Hann *et al*. [18] (*cf.* Figure S1 in Text S1, supplementary material).
- Protonation states and formal charges were set according to MOE's washing routine (carboxylic acids were deprotonated; most of the primary, secondary and tertiary amines were protonated).
- Duplicate entries were removed.

In the second step, the filtered compound set was subjected to a collection of preprocessing reactions. A set of 15 functional group addition (FGA) and functional group interconversion (FGI) reactions was compiled from the literature and encoded as Reaction-MQL expressions (for a complete list of preprocessing reactions see Table S2 in Text S1, supplementary material). FGA and FGI reactions are supposed to introduce reactive functional groups to building blocks to make them applicable to coupling reactions during the virtual compound construction process. Each time a building block was converted by any of the 15 reactions its original version was kept, and the converted building block was added to the library.

The third and final step of the preparation process comprises the annotation of reactive substructures (*i.e.* which building block can act as a reactant for which reaction). In order to be annotated as a reactant for a reaction, a building block has to match one of the reactant's substructure definitions exactly once. Forbidding the same functional group to be present multiple times is supposed to avoid unwanted side products or the need for excessive use of protecting groups in the actual chemical synthesis. (Please note that in the current version of the software no additional effort is made to estimate the reactivity of competing functional groups.) After annotation, building blocks are stored in a MySQL (Oracle Corporation, 500 Oracle Parkway, Redwood Shores, CA 94065, USA) database. The resulting building block library accessible to DOGS contains 25'144 entries.

### Construction Algorithm

**Figure 2:**
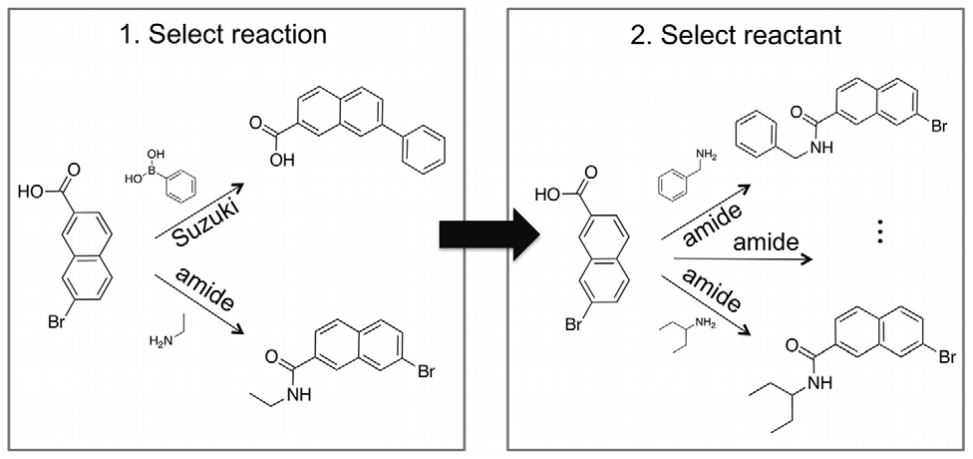
Two-step procedure of an extension cycle. *Step 1* (*left*) selects the reaction by scoring generated dummy products. In the example, only two reactions can be applied (Suzuki coupling and amide coupling), and the amide dummy product scores favorable. In *Step 2* (*right*), all reactants from the building block library exhibiting a suitable amine are added to the growing molecule *via* amide bond formation. The top-scoring product represents the extended intermediate product and is selected for the next design cycle.

DOGS generates new molecules by iterative fragment assembly. The design cycle comprises the modification of a current intermediate product by applying one of the chemical reactions from the library, *i.e.* the extension of the intermediate product (growing step). The product of a design cycle is an intermediate compound, which is modified in the subsequent iteration. A design cycle features two steps:

**Step 1: Selection of the applied reaction.** An intermediate product *Z* will typically exhibit more than one functional group that can be addressed by reactions from the reaction library. Each of these groups can potentially serve as an *attachment point* (AP) to connect another building block. In order to identify the most promising AP of *Z* and the reaction to apply, we used *minimal dummy fragments*. A minimal dummy fragment is a virtual molecule that exclusively features the minimal structural demands that have to be fulfilled in order to participate in a certain reaction. This concept is supposed to estimate the smallest structural changes a reaction will introduce (Figure 2). A one-component reaction does not define any minimal dummy fragment. It can directly be applied to a molecule without the involvement of a second variable reactant contributing any atom to the formed product. Thus, structural changes to *Z* do not need to be estimated but are determined by simply applying the reaction. In contrast, a two-component reaction defines two minimal dummy fragments.
For extending an intermediate compound *Z*, the algorithm first detects which of the implemented reactions can be applied to the attachment points offered by *Z*. Each of these reactions is applied to *Z* with a complementary minimal dummy fragment, resulting in a list of *dummy products*. Here, one dummy product corresponds to exactly one reaction. By subsequently scoring the dummy products, DOGS implicitly scores the corresponding reactions. The reaction yielding the top-scoring dummy product is pursued in Step 2. In case more than one top-scoring reaction is identified all of them are considered in Step 2.
**Step 2: Selection of a new building block.** In case Step 1 selected a one-component reaction, *Z* is directly modified. Otherwise (two-component reaction), the reaction is performed using all building blocks from the library holding the respective reactive substructure (Figure 2). Each generated product is evaluated according to the scoring function. The top-scoring compound is selected and represents the extended intermediate product for the next design cycle. If more than one intermediate product is scored favorable, all of them will be considered for the next round. In order to restrict the number of molecules generated during each step and to prevent combinatorial explosion, the maximal number of intermediate products proceeding to the next extension round was limited to 10.

The algorithm evaluates every building block processed by the dummy reaction steps according to the scoring function. Each of the *n* top-scoring building blocks is considered as a potential starting point for a distinct synthesis pathway. Parameter *n* is defined by the user and controls the number of compounds resulting from a design run.

Once the design of a new compound based on a selected starting building block is initiated it will be continued until one of two stop criteria is fulfilled.

The first stop criterion controls the molecular mass of the designed compounds. The reference compound's mass (100%) defines a relative lower (70%) and upper (130%) bound. A constructed molecule has to exhibit a molecular mass lying within these boundaries to be accepted as a valid final product. During the design of a new molecule the algorithm continuously adds building blocks until the constructed intermediate product exceeds the lower mass boundary. Up to this step the extension of the intermediate product is accepted even if its score value decreases. Once the molecular mass of the intermediate product exceeds the lower mass boundary, the algorithm will only accept a subsequent extension step if it leads to an improved score. In case the addition of a building block leads to a lower score or causes the molecular mass to exceed the upper mass limit, the last reaction step is neglected and the previous intermediate product is added to the list of final products.

**Figure 3:**
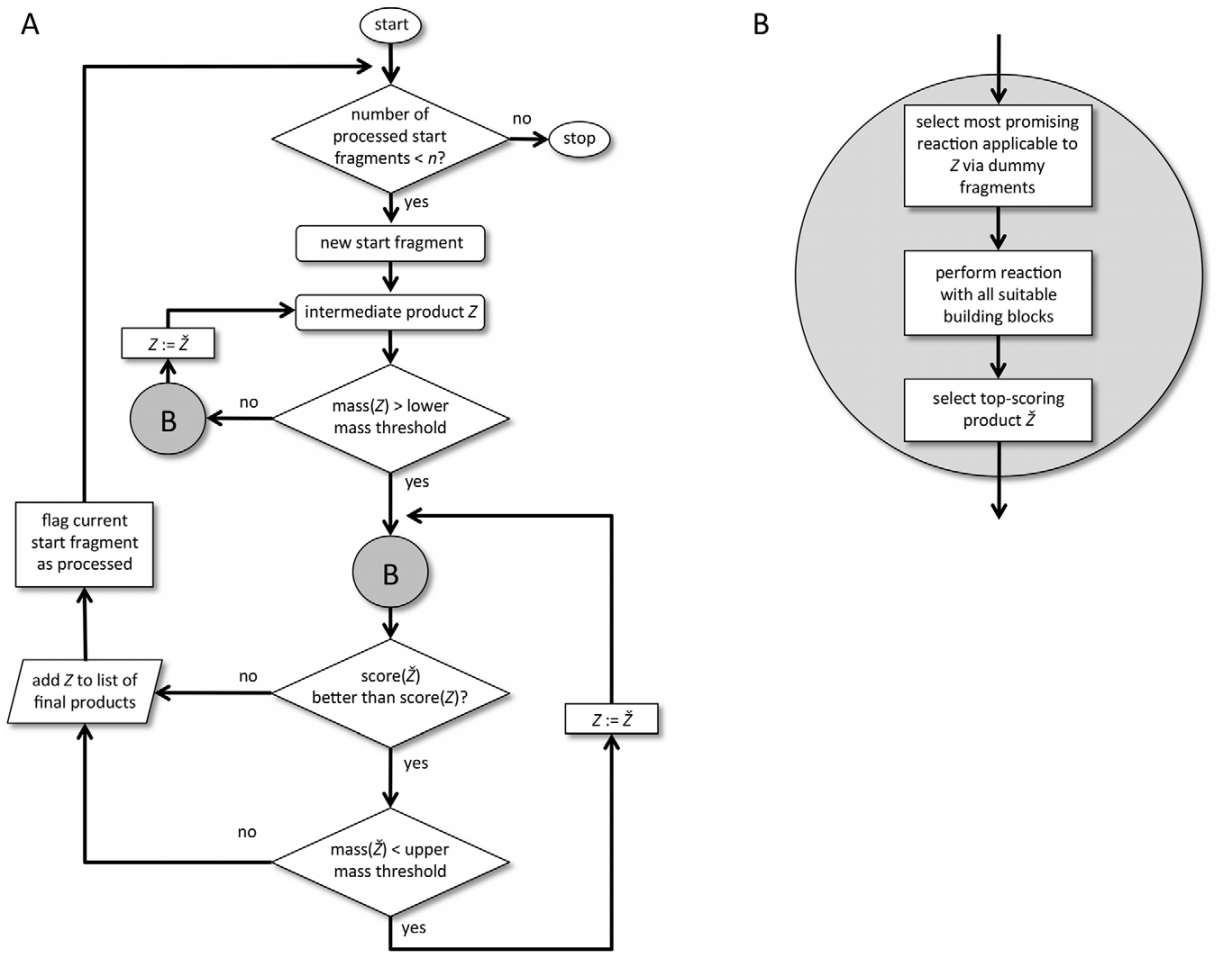
Flowchart of the molecule design algorithm. (**A**) The stop criterion controlling the maximum number of reaction steps is excluded from the flowchart for simplification. (**B**) Detailed description of flowchart element B (grey circle). It comprises the key steps taken to extend intermediate product *Z* and yield the top-scored intermediate product *Ž* ( = *Z* grown by an additional fragment) by applying *in silico* reactions.

The second stop criterion is supposed to truncate the number of synthesis steps to keep proposed synthesis pathways short. A pathway is interrupted regardless of any other condition when it exceeds a user-defined maximal number of synthesis steps (set to a value of four steps in all runs presented in this study). In this case, the intermediate product formed by the last valid reaction step is added to the list of final products, and a new synthesis pathway is initiated based on another starting building block. Figure 3 presents the core of the DOGS compound design algorithm.

DOGS tries to construct at least one compound starting from each of the *n* building blocks considered to be the most promising starting points. It is possible that an initiated synthesis path does not produce a final product. This happens when the growing intermediate product does not offer an attachment point to add another building block before it exceeds the minimal mass limit. In such a case, DOGS automatically skips this particular synthesis and increments *n* by 1 to guarantee that at least *n* final products are generated. Typically, a run will result in more than *n* final products because synthesis pathways can split if more than one top-scoring intermediate product is generated. In this case, multiple final products will be designed on the basis of a starting building block. All steps of the design algorithm are deterministic, *i.e.* two runs of DOGS with identical parameters will deliver identical results.

### Scoring Function

The scoring function assesses the quality of a molecule with respect to the design objective. Products of each stage of a virtual synthesis pathway (dummy products, intermediate products, final products) are evaluated by the same scoring function. DOGS employs a two-dimensional (2D) graph kernel method (ISOAK [19]) for scoring the designed molecules. The graph kernel was originally developed for similarity searching in virtual screening of compound databases, where it has been applied successfully [20]. ISOAK can be readily employed as a scoring function for ligand-based *de novo* design, where, like in virtual screening, similarity to a given reference ligand (a known bioactive compound) forms the key objective.

Briefly, ISOAK computes similarity values for two molecules based on their 2D topological structures. Molecules are interpreted as graphs, where atoms are represented as vertices and covalent bonds as edges between vertices (*molecular graph*). Hydrogen atoms are removed from the graph. Vertices are ‘colored’ by one of eight pharmacophoric feature types assigned to the corresponding atom (A: hydrogen-bond acceptor, D: hydrogen-bond donor, E: hydrogen-bond donor & acceptor, P: positive charge, N: negative charge, R: aromatic, L: lipophilic, 0: no type; the list of atom type definitions can be found in Table S3 in Text S1, supplementary material). A recursive definition of similarity between compared atoms (“two atoms are similar if their neighbors are similar”) is iteratively employed until the process converges. Parameter α controls the influence of the graph neighborhood, where higher values increase the impact of the neighborhood. Based on calculated atom-pair similarities, an optimal assignment of each atom of the smaller graph to one atom of the larger graph is computed. The assignment maximizes the sum of atom-pair similarities, which gives the overall similarity of the compared molecules. Similarity values are adjusted for compound size by scaling by the number of non-hydrogen atoms.

### Reduced Graph Representation

**Figure 4:**
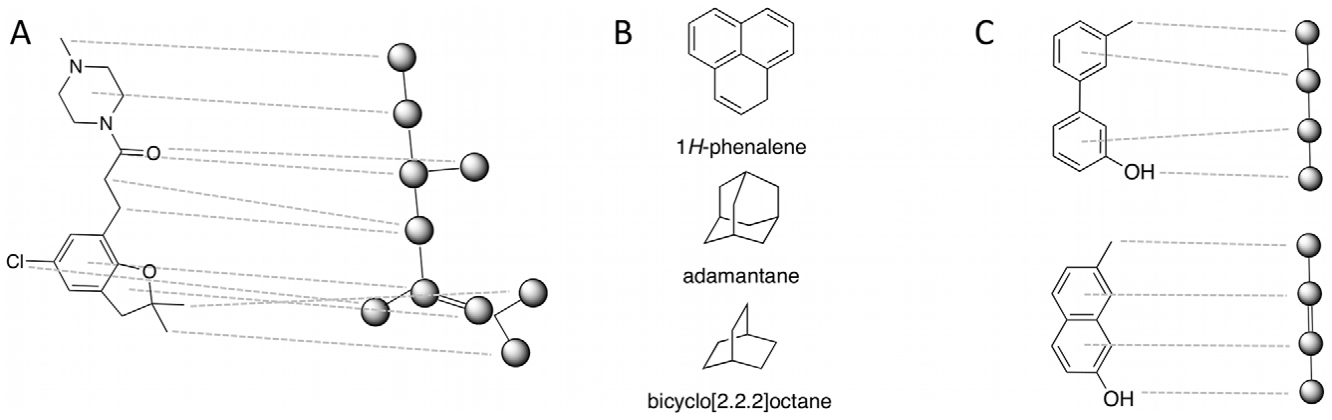
Reduced graph representation. (**A**) An example of a reduced graph representation. Dashed lines connect atoms or rings of the molecule (*left*) with their corresponding vertex of the reduced graph (*right*). For clarity only some lines are shown. (**B**) Examples of polycyclic (‘amalgamated’) substructures translated to a single vertex in the reduced graph. (**C**) Edges of order two are used to connect fused rings (*bottom*) in order to distinguish the shown cases of neighbored rings in reduced graph representation.

In addition to the molecular graph described in the previous section, a *reduced graph* representation of molecules was implemented as an alternative description of molecules. Reduced graphs only represent the overall topological arrangement of structural features. The motivation to use them for *de novo* design was to encode molecules in a representation featuring a higher level of abstraction from the molecular composition and constitution. Similar to the FeatureTrees [21] approach, the reduced graph representation employed by DOGS reduces cyclic substructures as well as clusters of ‘lipophilic’ and ‘no type’ atoms to single vertices (Figure 4A). In general, each ring that is part of the smallest set of smallest rings (SSSR [22]) is converted to one vertex. Exceptions of this rule are fused ring systems with atoms belonging to more than two rings of the SSSR. In this case, it is not possible to represent each ring as a single vertex and still obtain a simplified acyclic graph representation of the molecule. Such ‘amalgamated’ ring systems are reduced to a single vertex as a whole (Figure 4B). In order to distinguish the reduced graph representation of two adjacent rings that are connected by a bond and two fused rings (rings sharing atoms), the corresponding vertices of reduced graphs representing the rings are connected by an edge of order one (‘single bond’) in the former case and two (‘double bond’) in the latter (Figure 4C).

Vertices of reduced graphs are labeled with bit vectors that store information about the atoms they represent. These bit vectors consist of ten bits (one for each of the eight atom types, and two additional bits standing for ‘ring’ and ‘amalgamated ring system’, respectively). A vertex bit is set if the corresponding feature is present in the set of atoms the vertex encodes. Vertices not only store the bit vector but also the number of atoms they represent. Accordingly, a benzene substructure would be converted to a single vertex which is labeled by a bit vector with bits for ‘ring’ and ‘aromatic’ set to 1, and stores an atom count of six. Pyridine would be encoded in the same way, except for the bit ‘hydrogen-bond acceptor’ being also set to 1.

Bit vectors (*bv*) and atom counts (*ac*) are used to compute the similarity of two vertices *A* and *B* of reduced molecular graphs. The similarity is computed by multiplying two terms (Eq. 1).
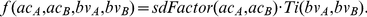
(1)Term 1 (*sdFactor*) returns a value between 0 and 1 depending on the difference between the atom count values of compared vertices (Eq. 2), defined as
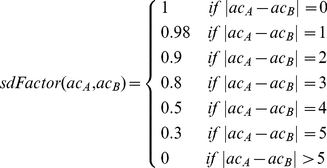
(2)Term 2 (*Ti*) is the Tanimoto index for bit vector comparison (Eq. 3).
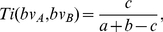
(3)where *c* is the number of bits set to 1 in both vectors, *a* is the number of bits set to 1 in *bv_A_* and *b* is the number of bits set to 1 in *bv_B_*. Component *sdFactor* can be seen as a penalty function for atom count differences modulating the Tanimoto index. In case the atom count of compared vertices is equal (*e.g.* two six-membered rings are compared), *f_vc_* reduces to the Tanimoto index. If the difference between the atom counts exceeds five, *f_vc_* will return 0 regardless of the calculated *Ti* for the bit vectors.

All other components of ISOAK including the edge comparison are identical to the molecular graph comparison. ISOAK can only processes graphs with a maximum vertex connectivity of six, *i.e.* a vertex of a graph processed by ISOAK must not have more than six directly connected neighbors. While this will not happen in molecular graphs (typically, no element that is present in drug-like molecules will form more than six covalent bonds), such cases can occur in reduced graphs. For example, 1*H*-phenalene (Figure 4B) is represented as a single vertex and offers up to nine positions for substitution. Molecules containing vertices with more than six neighbors in their reduced graph representation are excluded from subsequent steps and discarded.

The molecular representation used in a design run is selected by the user, *i.e.* a DOGS run is either based on the molecular graph or reduced graph scoring scheme.

### Implementation

The DOGS software was implemented in the programming language Java (Oracle Corporation, 500 Oracle Parkway, Redwood Shores, CA 94065, USA) version 1.6 and uses the Chemistry Development Kit (CDK, version 1.0.2) [23], [24].

## Results/Discussion

### Theoretical Analysis: Design of Potential Trypsin Inhibitors

**Figure 5:**
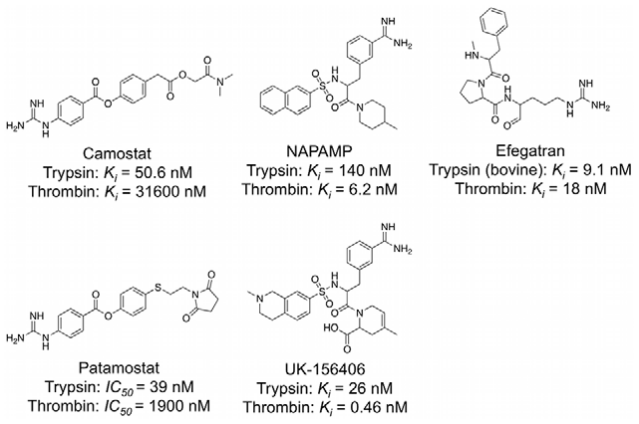
Known trypsin inhibitors. Five trypsin inhibitors served as reference compounds for DOGS design runs (Camostat [45], NAPAMP [46], Efegatran [47], Patamostat [48], [49], UK-156406 [50]).

Our initial theoretical analyses of the algorithm were based on *de novo* designed compounds originating from ten distinct DOGS runs. Five trypsin inhibitors served as reference ligands for these runs (Figure 5). For each reference, a design run based on the molecular graph representation (α = 0.875, default of ISOAK) and a second run applying the reduced graph representation (α = 0.4, selected based on preliminary empiricism) was performed. The number of start fragments was set to 200. The ten runs resulted in a total of 1'767 unique compounds.

#### Molecular properties

**Figure 6:**
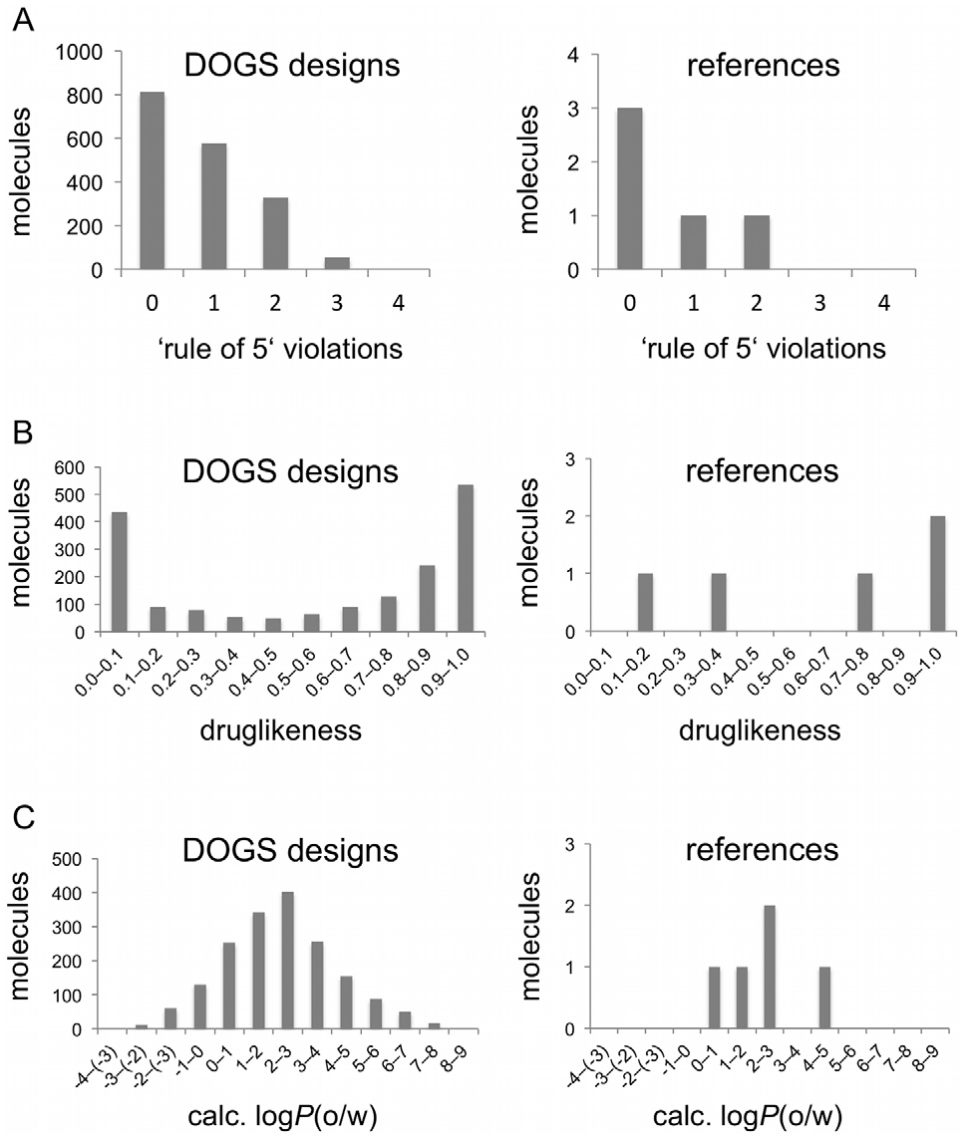
Property distributions. Comparison of property distributions between compounds designed by DOGS (*left*) and the reference compounds (*right*). ‘Rule of 5’ violations (**A**) and log*P*(o/w) values (**C**) were calculated using MOE. Dug-likeness scores (**B**) were computed by a trained neural network classifier (1 = high drug-likeness).

Although successful *de novo* design will likely be followed-up by structural optimization of selected compounds in order to improve their potency and pharmacokinetic properties, the computer-designed compounds are supposed to already have drug-like properties in the first place. In order to assess the drug-likeness of molecules generated by DOGS, violations of Lipinski's ‘rule of 5’ [25] were recorded for the 1'767 molecules using a descriptor implemented in the software MOE. An analysis of the Lipinski ‘rule’ violations revealed that most of the compounds (79%) constructed by DOGS violate less than two rules (Figure 6A). Only 52 proposed molecules (3%) cause three violations. The distribution of designed compounds mirrors the one of the reference ligands. A second analysis of the drug-likeness of DOGS designs was carried out for the same set of designed compounds using an artificial neural network [26]. This classifier was trained on a set of drugs and presumed non-drugs to score molecules between 0 (low drug-likeness) and 1 (high drug-likeness). Out of the 1'767 molecules designed by DOGS 904 (51%) receive a score of 0.8 or higher (Figure 6B). A considerable number (436) of the DOGS molecules receive a poor drug-likeness score below 0.1. This can probably be explained by the fact that one of the reference compounds receives a low drug-likeness score (Patamostat, *score* = 0.11). Compounds designed to maximize similarity to this reference can be expected to receive poor drug-likeness scores as well.

Lipophilicity is considered a relevant physicochemical property for drug candidate molecules [27]. A common parameter closely related to lipophilicity is the octanol-water partition coefficient (log*P*(o/w)) [28]. One of the Lipinski guidelines states that a log*P*(o/w) value greater than five decreases the potential of a molecule to be absorbed [27]. Log*P*(o/w) values were calculated for the five trypsin reference ligands and the molecules designed by DOGS using the log*P*(o/w) descriptor implemented in MOE (Figure 6C). The distribution of calculated log*P*(o/w) values of DOGS designs approximates a unimodal distribution centered at 2<log*P*<3. This is in agreement with the distribution of values calculated for the reference ligands. Apparently, DOGS is able to mimic this property of the references in the generated compounds, although it is not explicitly considered during the design.

It is of critical importance that molecules designed *in silico* not only exhibit some desired properties but also are amenable to chemical synthesis in order to be of any practical value for drug discovery projects. A molecular descriptor (*rsynth*) implemented in the software package MOE estimates the ‘synthesizability’ of molecules as the fraction of heavy atoms that can be traced back to starting material fragments resulting from retrosynthetic rules. A score value of 1 means full coverage of atoms and expected high synthesizability. The *rsynth* descriptor was calculated for both the reference set and the set of *de novo* designed molecules. Accordingly, most of the DOGS designs are deemed synthesizable, as 77% of compounds receive a score greater than 0.9. Most of the remaining designs receive scores between 0.4 and 0.8. Reference compound UK-156406 was scored low (*rsynth* = 0.37). A total of 36% (141 of 397) of all DOGS designs scoring below 0.8 originate from this reference ligand, which exceeds an expected fraction of 20% assuming that low-scoring designs are derived from all five references to equal parts. This means that low synthesizability scores are enriched for molecules originating from a reference compound that is scored unfavorably. This finding points to a positive correlation between the *rsynth* score of a reference compound and *rsynth* scores of derived DOGS compounds, probably due to the principle of structural similarity underlying the scoring scheme. However, a larger number of examples will be needed to support this hypothesis on a solid statistical basis. For each of the five trypsin reference ligands, we found a consistent trend that design runs based on the molecular graph representation yield slightly higher averaged *rsynth* scores than the corresponding runs using the reduced graph representation (*cf.* Table S4 and Figure S2 in Text S1, supplementary material). Overall, this preliminary result may be considered a success of the DOGS approach to generate molecules that are deemed highly synthesizable.

In summary, the majority of the DOGS designs possesses drug-like properties and is chemically plausible. Most compounds are deemed being amenable to chemical synthesis. The proposed molecules resemble the reference compounds in properties that are not explicitly considered by the scoring function.

#### Bioisosteric replacement

**Figure 7:**
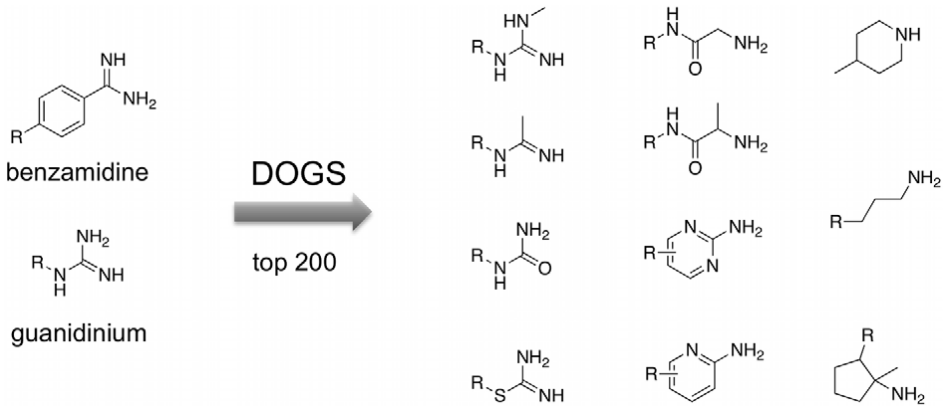
Bioisosteric replacement. Side-chains addressing the S1 pocket present in the reference compounds (*left*) and surrogates suggested by DOGS found in top-scored 200 designs (*right*).

Bioisosteric replacement [29] of functional groups is key to successful *de novo* design. In order to test DOGS for its ability to perform bioisosteric replacement, the list of 1'767 potential trypsin ligands designed by the software (resulting from ten runs based on five trypsin inhibitor references) was ranked according to the scores assigned by DOGS. The top 200 molecules were analyzed for functional groups that replace side-chains of the reference compounds addressing the S1 pocket of the enzyme (guanidinium and benzamidine, Figure 7).

**Figure 8:**
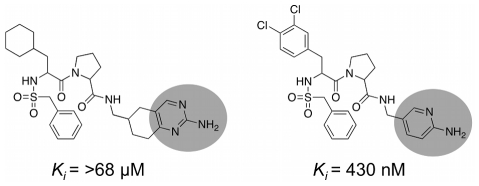
Side-chains addressing the S1 pocket of trypsin. Known inhibitors of trypsin exhibiting pyrimidin-2-amine [51] (*left*) and the pyridin-2-amine [52] (*right*) side-chains (grey circles). These moieties were also suggested by DOGS as bioisosters for side-chains of the reference ligands addressing the S1 pocket of trypsin.

Starting at rank position 78 (compounds on higher ranks exhibit one of the fragments present in the references), DOGS suggested eleven different side-chains replacing the reference fragments. Most of them offer the possibility to interact with the negatively charged aspartate side-chain of the S1 binding pocket of trypsin by a positively ionizable nitrogen atom. The terminal urea group and the two aromatic fragments (pyrimidin-2-amine and pyridin-2-amine) are exceptions, where the nitrogen will likely not carry a positive charge. The formation of this salt bridge is a known key interaction inside the S1 pocket [30]. Although salt bridge formation is unlikely for these three fragments, they might still be able to form a hydrogen-bond to the aspartate side-chain. In fact, both pyrimidin-2-amine and pyridin-2-amine act as S1-addressing moieties in known trypsin inhibitors (Figure 8). In addition, the list of proposed side-chains contains an alkyl chain carrying a terminal nitrogen atom. This fragment resembles the side-chain of lysine, which is part of the substrates occupying the S1 pocket during peptide bond cleavage [30].

In summary, DOGS was able to suggest reasonable bioisosters for parts of the reference ligands addressing the S1 pocket of trypsin, including experimentally validated examples.

#### Examples of designed compounds

**Figure 9:**
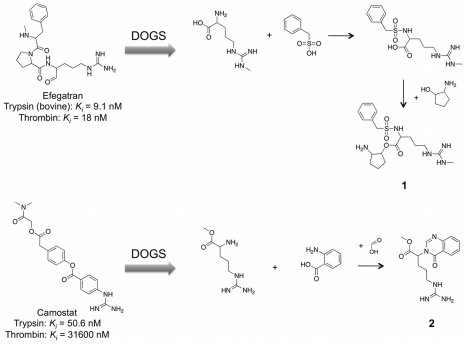
Suggested trypsin inhibitors. Compounds **1** and **2** were proposed by the software as potential trypsin inhibitors. Reference ligands (Efegatran [47], Camostat [45]) and suggested synthesis pathways are presented for both candidate structures.

Two examples selected from the list of structures proposed by DOGS as potential trypsin inhibitors are presented in Figure 9. Compounds **1** and **2** were obtained from design runs based on Efegatran and Camostat. Compound **1** (originating from the reference ligand Efegatran) features a central sulfonamide moiety that is not present in the reference molecule. In this example, DOGS replaced a substructure of the reference by a structurally different but presumably isofunctional fragment that is found in other trypsin inhibitors (for example in NAPAMP and UK-156406, Figure 5). The guanidinium side-chain of Efegatran was exchanged by the structural analog 3-methylguanidinium. The overall composition of functional groups in compound **1** resembles the topological arrangement in the reference. The synthesis route proposed by DOGS will probably have to be augmented by the use of protection groups. For example, the formation of the ester bond in the last synthesis step can be disturbed by the competing formation of an amide bond with the primary amine of reactant 2-aminocyclopentanol. Protection of the amine group could solve this problem. Note that DOGS currently does not consider protection groups. Competing side reactions are only addressed by avoiding multiple occurrences of the same functional group in a reactant.

Compound **2** was derived using Camostat as reference ligand. Compared with the former example of molecule **1**, computer-generated molecule **2** is structurally more distinct from its reference. While the guanidinium group of the reference is preserved, it is connected to an alkyl chain instead of a phenyl ring. Alkyl linkers connecting the guanidinium group can also be found in Efegatran and in the side-chain of arginine, a natural substrate of the trypsin S1 pocket [30]. An aromatic substructure distant from the part addressing the S1 pocket is another feature of compound **2** that can be found in known trypsin ligands as well (*cf.* NAPAMP, Figure 5). As the main goal of *de novo* design is the generation of isofunctional but structurally novel molecules, compound **2** might be considered a potential candidate for further investigations.

### Prospective Study 1: γ-Secretase

DOGS was employed to propose candidate structures as new modulators of γ-secretase, an aspartic protease that cleaves the amyloid precursor protein (APP) and generates potentially toxic amyloid-β (Aβ) peptides [31]. Formation and accumulation of soluble Aβ oligomers in the brain is thought to be a primary pathological event in Alzheimer's disease [32]. γ-Secretase modulators shift the product ratio of APP processing from the highly amyloidogenic Aβ42 peptides towards shorter Aβ fragments with a lower propensity to aggregate like Aβ38 [31], [33].

**Figure 10:**
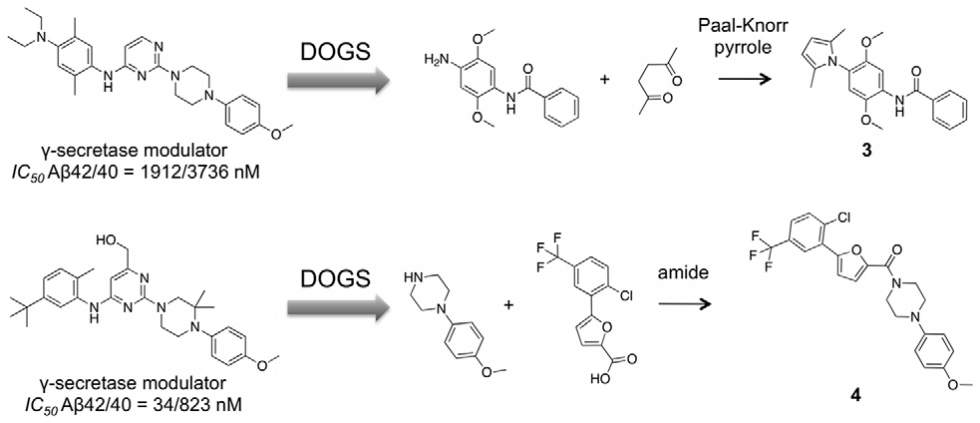
Automated design of γ-secretase modulators. Compounds **3** and **4** were proposed by DOGS as potential modulators of γ-secretase. Synthesis plans were suggested by the software and successfully pursued. Molecules **3** and **4** originate from distinct runs based on different reference ligands [53].

Four different reference ligands known to modulate γ-secretase were selected. For each reference compound, two DOGS runs (molecular graph representation, α = 0.875; reduced graph representation, α = 0.4) were performed. The resulting eight compound lists were visually inspected, and two appealing ligand candidates **3** and **4** were selected for synthesis (Figure 10).

Synthesis plans were readily traceable as suggested by the software. One-step reactions yielded the desired products in both cases. Hence, DOGS demonstrated its ability to come up with compounds considered as promising candidates by medicinal chemists and proved to be chemically accessible as suggested (*cf*. Figure S3, Figure S4, Protocol S1 and Protocol S2 in Text S1, supplementary material). Synthesized compounds were tested for their ability to modulate the γ-secretase product spectrum as previously described [34]. CHO cells with stable overexpression of human APP and presenilin-1 were treated with increasing concentrations of **3** and **4**. Subsequently, concentrations of secreted Aβ peptides were detected in cell supernatants by sandwich ELISA using C-terminus specific antibodies that distinguish between Aβ38, Aβ40, and Aβ42 peptide species [34]. ELISA results indicate inverse modulation of γ-secretase activity (*cf*. Figure S5 in Text S1, supplementary material). Compound **3** induced a dose-dependent increase in Aβ42 levels with a concomitant decrease in Aβ38 levels. Similar results were obtained for compound **4**. This pattern of inverse γ-secretase modulation has previously been observed, *e.g.* with derivatives of the non-steroidal anti-inflammatory drug indomethacin [35]. Although inverse γ-secretase modulation is not the effect intended for potential treatment of Alzheimer's disease, these results clearly show that DOGS is able to design compounds with pharmacological activity on the macromolecular target. Compounds **3** and **4** can serve as tool compounds and – more importantly – as starting points for an optimization of the pharmacological profile by structural modification.

### Prospective Study 2: Human Histamine H_4_ Receptor

Histamine is a biogenic amine involved in a plethora of signaling pathways as a messenger. Four subtypes of histamine receptors (*h*H_1_R – *h*H_4_R) are known in human. All subtypes belong to class A (rhodopsin-like) of the G-protein coupled receptor (GPCR) superfamily [35], [36]. Some antagonists of *h*H_1_R and *h*H_2_R are approved drugs for the treatment of allergic reactions and ulcer. Clinical trials of *h*H_3_R antagonists for the therapy of diseases of the central nervous system, such as epilepsy, schizophrenia and sleep/wake disorders are currently in progress [37].

**Figure 11:**
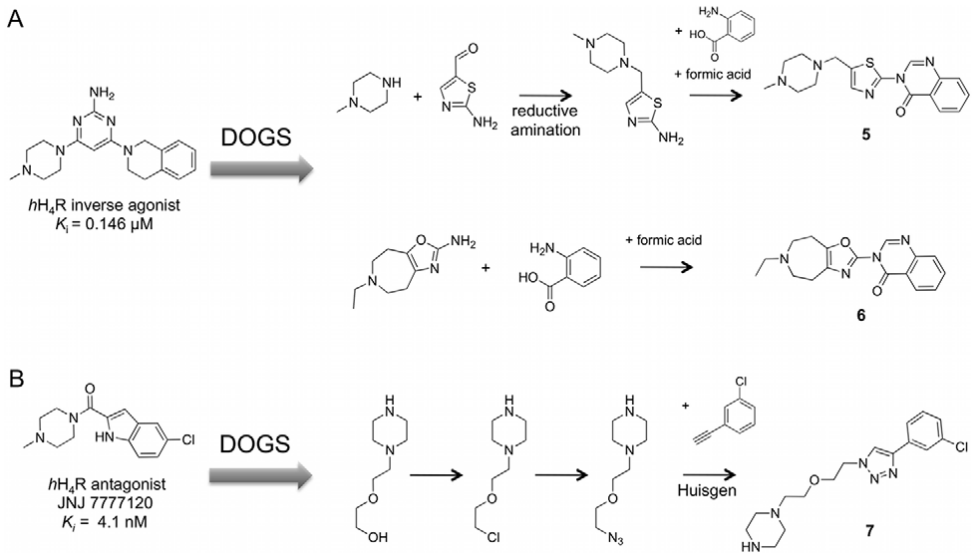
Automated design of H_4_ ligands. Compounds **5** and **6** were proposed by DOGS based on an inverse agonist of *h*H_4_R [40] (**A**). Compound **7** is a design originating from the *h*H_4_R antagonist JNJ7777120 [54] (**B**).

We applied DOGS to provide ideas for new selective antagonists or inverse agonists of *h*H_4_R. For this purpose, two reference ligands (an inverse agonist and an antagonist) were employed (Figure 11). For each reference, the molecular graph representation (α = 0.875) as well as the reduced graph representation (α = 0.4) was applied, resulting in a total of four DOGS runs. Three prioritized designs **5**–**7** are presented in Figure 11.

*N*-methylpiperazine is present in both reference compounds. This moiety is often used as a basic head group in H_4_ receptor ligands [38]. The positive charge of basic amines is believed to form a key interaction to a negatively charged amino acid side-chain of the protein [39]. While in compound **5** the *N*-methylpiperazine moiety is preserved, it is replaced in **6** and **7** by isofunctional groups. Both represent aliphatic rings with basic nitrogen atoms, which provide a chance to undergo the charge-mediated interaction with the receptor. Localization of aromatic ring systems of the reference compounds is also approximately kept within the proposed structures.

The attempt to follow the synthesis scheme proposed for compound **5** was not continued after facing solubility problems of the aminothiazole building block, which led to extremely poor yields of the intermediate product. Awkward behavior of reactant building blocks represents one potential problem of the transition from *in silico* to bench synthesis, illustrating the demand of this endeavor.

**Figure 12:**
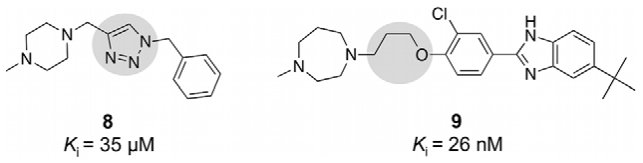
Structural features of H_4_R ligands. Highlighted features of known H_4_R ligands (compound **8** [40]: central triazole ring; compound **9** [41]: ether alkyl linker) are combined in designed compound **7**. None of these features is present in the reference ligand underlying the design of **7**.

Compound **7** was deemed to be of special interest, as it combines two structural elements that can be found in reported H_4_R ligands: an alkylic linker chain with an ether bridge and a central triazole ring (Figure 12). Notably, both structural elements are absent from the reference compound. The moderate affinity of the triazole-carrying ligand **8** (*K*_i_ = 35 µM) [40] may be caused by a missing hydrogen-bond acceptor in the central part, an interaction site that is believed to play a role in ligand binding to H_4_R [39]. The oxygen atom of the ether bridge present in designed compound **7** and H_4_R ligand **9** [41] is able to act as a hydrogen-bond acceptor. The ISOAK scoring function of DOGS assigns this oxygen to the carbonyl oxygen of the reference, which also represents a hydrogen-bond acceptor.

In order to test for the hypothesis that a combination of the features – as found in compound **7** – might lead to *h*H_4_R affinity, compound **7** was selected for synthesis and testing. The synthetic procedure was realized exactly as suggested by the software (*cf.* Figure S6, Protocol S3 in Text S1, supplementary material). Binding affinity of compound **7** was determined in a competitive binding assay by measuring displacement of radioactively labeled [^3^H]histamine bound to *h*H_4_R [42]. Membrane preparations of insect Sf9 cells expressing *h*H_4_R together with G-protein subunits Gα_i2_ and Gβ_1_γ_2_ were performed to yield the protein. A similar assay was used to measure the activity on *h*H_3_R (reference ligand: [^3^H]*N* ^α^-methylhistamine) [43]. Compound **7** exhibits only very weak affinity to *h*H_4_R. From three measurements, a mean *K*_i_ of 436±137 µM was determined. Comparable results were found for the activity of **7** on the *h*H_3_R receptor (*K*_i_ = 466±209 µM, averaged over four distinct tests).

A reason for the weak affinity of **7** might be a missing hydrogen-bond donor in the central part, which has been suggested to play a role in the interaction of some known H_4_ ligands with the receptor [39], [44]. In fact, the nitrogen atom of the indole moiety of reference compound JNJ7777120 can act as a hydrogen-bond donor. Introduction of a hydrogen-bond donor to the central part and the exchange of the piperazine head group against *N*-methylpiperazine represent comparably small structural changes to compound **7** and might be considered as first steps to improve binding affinity.

Additionally, compound **7** was tested against a panel of 30 other human GPCRs (assays were performed by Cerep, Le bois l'Evêque, 86600 Celle l'Evescault, France; human GPCRs tested: A_2A_, A_2B_, A_3_, α_1A_, α_1B_, α_2C_, β_1_, β_2_, CCK_1_ (CCK_A_), D_1_, D_3_, D_4.4_, H_1_, H_2_, M_1_, M_2_, M_3_, M_4_, M_5_, NK_1_, δ_2_ (DOP), κ (KOP), μ (MOP), 5-HT_1D_, 5-HT_2A_, 5-HT_2B_, 5-HT_2C_, 5-HT_4e_, 5-HT_6_, 5-HT_7_). Notably, an agonistic effect on the κ opioid receptor (21% of the effect of the reference agonist U50488, *EC*_50_ = 1.2 nM, *n* = 2), and antagonistic effects on the δ_2_ opioid receptor (76% residual activity of the receptor in the presence of the reference agonist naltrindole (*IC*_50_ = 0.37 nM, *n* = 2) and the 5-HT_1D_ receptor (62% residual activity of the receptor in the presence of the reference agonist methiothepin, *IC*_50_ = 1.1 µM, *n* = 2) were observed. For other GPCRs in the panel only weak responses in the single digit or low double-digit percent range were found. These findings suggest that, while lacking high affinity and selectivity to the primary target *h*H_4_R, compound apparently **7** features a general pharmacophore motif of aminergic GPCRs ligands.

Although the DOGS design approach is capable of suggesting compounds of practical relevance, a potential improvement to scoring would be to directly incorporate knowledge of a particular pharmacophore, *i.e.* the requirement for a particular spatial arrangement of potential interaction sites. This is only implicitly considered by the current scoring scheme, which can lead to high scores for designs exhibiting a spatial rearrangement of interaction sites. We therefore consider combining the design algorithm with scoring functions capable of taking 3D pharmacophore models into account in future versions of the software.

In conclusion, we present a detailed description of a new method for automated *de novo* design. The software had already shown its potential to suggest selective and potent new compounds together with a pursuable route to synthesize them in a previous study [14]. Here, we provide in-depth insight into the algorithm and analyze it theoretically. In addition, two prospective case studies on automated design of bioactive compounds are presented. An important feature of the algorithm is its minimal demand for prior knowledge about the biological target. A single reference compound is sufficient to have the algorithm come up with suggestions for active compounds. This feature might be of special merit for drug discovery addressing structurally unexplored targets. However, despite these advances generating innovative and patentable molecules with biological activity from scratch remains a demanding goal. Current software solutions to this problem are far away from being ‘click-and-harvest’ applications guaranteed to produce readily exploitable results. *De novo* design relies on the thoughtful intervention and support of a human expert. Nevertheless, it can be a valuable source of inspiration and new ideas for medicinal chemistry.

## Supporting Information

Text S1 Supplementary material comprises coupling reactions, preprocessing reactions, unwanted substructures, description of pharmacophore substructures, synthesis protocols and analytical data.
(PDF)
