# Supplementary material for: DOGS: Reaction-Driven de novo Design of Bioactive Compounds
Source: PLoS Comput Biol. 2012 Feb 16;8(2):e1002380. doi: 10.1371/journal.pcbi.1002380 (PMC3280956; doi:10.1371/journal.pcbi.1002380)
Supplement: Text S1 — Supplementary material comprises coupling reactions, preprocessing reactions, unwanted substructures, description of pharmacophore substructures, synthesis protocols and analytical data. (PDF) [file pcbi.1002380.s001.pdf]

# Supplementary Material

## "*DOGS: reaction-driven* de novo *design of bioactive compounds*"

Markus Hartenfeller[1], Heiko Zettl[1], Miriam Walter[2], Matthias Rupp[1], Felix Reisen[1], Ewgenij Proschak[2], Sascha Weggen[3], Holger Stark[2], Gisbert Schneider[1,*]

[1]Swiss Federal Institute of Technology (ETH), Department of Chemistry and Applied Biosciences, 8093 Zürich, Switzerland

[2]Goethe-University, Institute of Pharmaceutical Chemistry, LiFF/OSF/ZAFES, 60438 Frankfurt am Main, Germany

[3]Department of Neuropathology, Heinrich-Heine-University, 40225 Düsseldorf, Germany

[*]Email: gisbert.schneider@pharma.ethz.ch

**Table of contents**

| | page |
|---|---|
| Table S1. Coupling reactions | 2 |
| Table S2. Preprocessing reactions | 17 |
| Figure S1. Unwanted substructures | 19 |
| Table S3. Pharmacophore typing | 20 |
| Table S4. Synthetic accessibility score | 21 |
| Figure S2. Synthetic accessibility score | 21 |
| Figure S3. Synthesis of compound **3** | 22 |
| Protocol S1. Preparation of compound **3** | 22 |
| Figure S4. Synthesis of compound **4** | 22 |
| Protocol S2. Preparation of compound **4** | 22 |
| Figure S5. Modulation of γ-secretase activity | 23 |
| Figure S6. Synthesis of compound **7** | 24 |
| Protocol S3. Preparation of compound **7** | 24 |
| References | 24 |

**Table S1.** Coupling reactions. Each reaction is specified by (i) Reaction-MQL expression, (ii) schematic structural representation, (iii) minimal structure of educt(s) encoded as SMILES, also representing the dummy fragment used during construction (schematic representations serve as examples only and not necessarily correspond exactly to the minimal dummy compounds).

1. c$1:c4[allHydrogens=1]:c(-C6[allHydrogens=2]-C7[allHydrogens=2]-N2-C3(=O5)-C):c:c:c$:1 >> Bischler-Napieralski >> C6-C7-N2=C3-c4

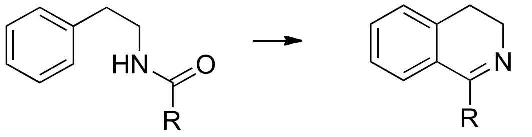


c1cc(CCNC(=O)C)ccc1

2. c$1:c4[allHydrogens=1]:c(-C6(-O8[allHydrogens=1])-C7[allHydrogens=2]-N2-C3(=O5)-C):c:c:c$:1 >> Pictet-Gams >> C6=C7-N2=C3-c4

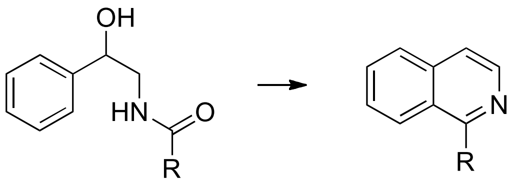


c1cc(C(O)CNC(=O)C)ccc1

3. c2[allHydrogens=1]:c(-C5[sp3 & !ring]-C6[sp3 & !ring]-N7[allHydrogens=2 & charge=0]):c[!bound(-H)] ++ C3[allHydrogens=1](=O4)-C >> Pictet-Spengler (charge 1) >> C5-C6-N7-C3-c2

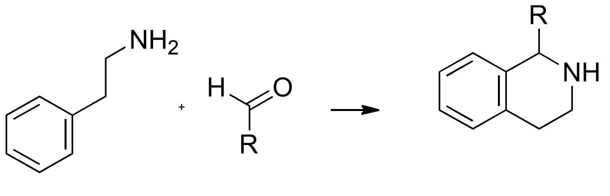


c1cc(CCN)c(C)cc1 + CC(=O)

c2[allHydrogens=1]:c(-C5[sp3 & !ring]-C6[sp3 & !ring]-N7[allHydrogens=3 & charge=1]):c[!bound(-H)] ++ C3[allHydrogens=1](=O4)-C >> Pictet-Spengler (charge 2) >> C5-C6-N7-C3-c2

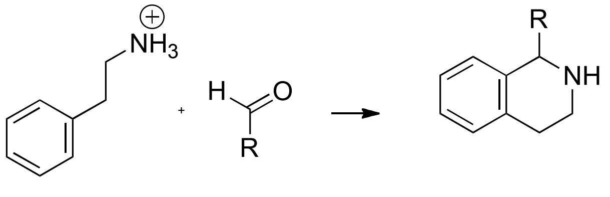


c1cc(CC[NH3+])c(C)cc1 + CC(=O)

4. c$1:c:c:c(-N3[allHydrogens=2]):c2[allHydrogens=1]:c$:1 ++ c-C4(=O5)-C6-*7[Cl|Br] >> Bischler Indole >> N3-C4=C6-C2

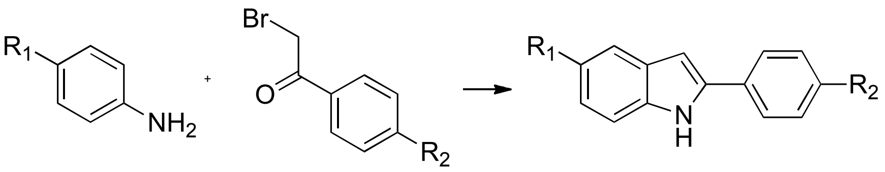

c1c(N)cccc1    +    c1c(C(=O)CBr)cccc1

5. c$1:c[allHydrogens=1]:c(-N7[allHydrogens=2]):c(-N8[bound(-H)]):c[allHydrogens=1]:c$:1 ++ C3(=O4)(-O5[allHydrogens=1])-C >> Benzimidazol (charge 1) >> N7=C3-N8

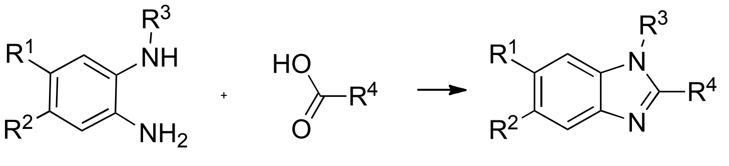

c1cccc(N)c1NC    +    CC(=O)O

c$1:c[allHydrogens=1]:c(-N7[allHydrogens=2]):c(-N8[bound(-H)]):c[allHydrogens=1]:c$:1 ++ C3(=O4)(-O5[charge=-1])-C >> Benzimidazol (charge 2) >> N7=C3-N8

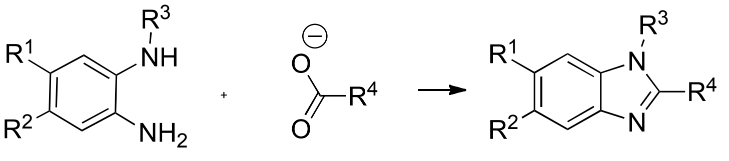

c1cccc(N)c1NC    +    CC(=O)[O-]

6. C-C4(-*5[Cl|Br])-C6(=O7)-C >> Aminothiazol >> C4$8-S-C(-N)=N-C6$=8

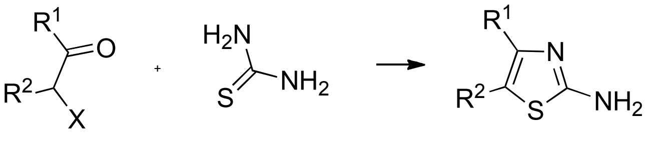

CC(=O)C(Br)C

7. c$1:c[allHydrogens=1]:c(-O7[allHydrogens=1]):c(-N8[allHydrogens=2]):c[allHydrogens=1]:c$:1 ++ C3(=O4)(-O5[allHydrogens=1])-C >> Benzoxazol (charge 1) >> O7-C3=N8

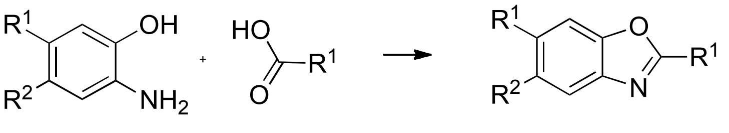

c1ccc(N)c(O)c1    +    CC(=O)O

c$1:c[allHydrogens=1]:c(-O7[allHydrogens=1]):c(-N8[allHydrogens=2]):c[allHydrogens=1]:c$:1 ++ C3(=O4)(-O5[charge=-1])-C >> Benzoxazol (charge 2) >> O7-C3=N8

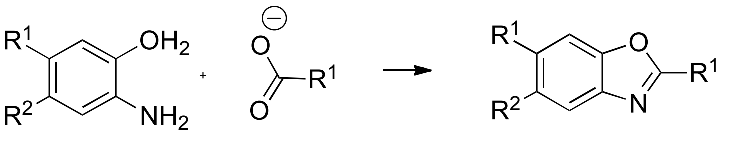

c1ccc(N)c(O)c1    +    CC(=O)[O-]

8. c$1:c:c(-S7[allHydrogens=1]):c(-N8[allHydrogens=2]):c:c$:1 ++ C3[allHydrogens=1](=O4)-c >> Benzothiazol >> S7-C3=N8

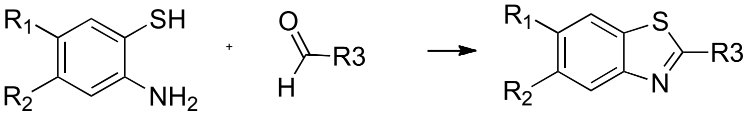


c1ccc(N)c(S)c1 + c1cc(C(=O))ccc1

9. c$1:c:c(-O7[allHydrogens=1]):c(-C8[allHydrogens=1]=O9):c:c$:1 ++ *5[Cl|Br]-C3[allHydrogens=2]-C(=O)-C >> Rap-Stoermer >> O7-C3=C8

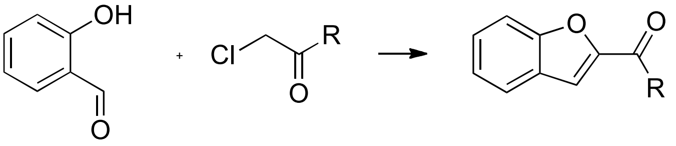


c1ccc(O)c(C(=O))c1 + CC(=O)CCl

10. c$1:c:c(-N1[allHydrogens=2]):c(-C2(=O)-O4[allHydrogens=1]):c:c$:1 >> Niementowski (charge 1) >> C2-N-C=N1

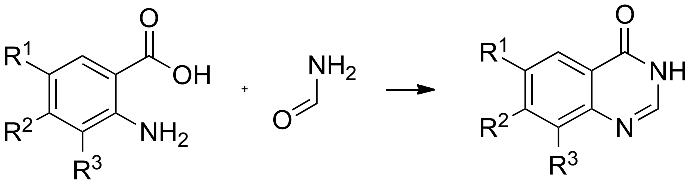


c1ccc(N)c(C(=O)O)c1

c$1:c:c(-N1[allHydrogens=2]):c(-C2(=O)-O4[charge=-1]):c:c$:1 >> classical Niementowski (charge 2) >> C2-N-C=N1

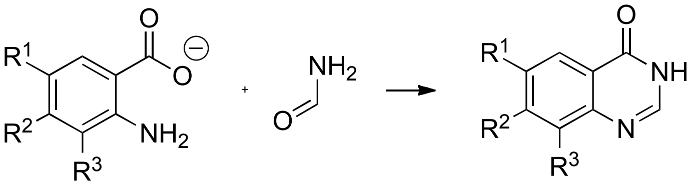


c1ccc(N)c(C(=O)[O-])c1

11. c[allHydrogens=1]$1:c[allHydrogens=1]:c(-N2[allHydrogens=2]):c(-C5(=O)-O4[allHydrogens=1]):c[allHydrogens=1]:c[allHydrogens=1]$:1 ++ C-N3[allHydrogens=2 & charge=0] >> Quinazolinone (Ladung 1) >> N2=C-N3-C5

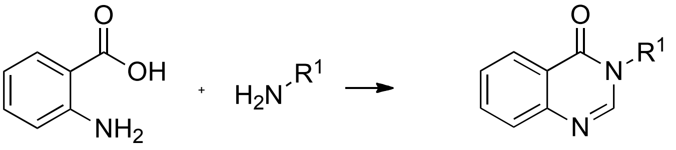


c1ccc(N)c(C(=O)O)c1 + CN

c[allHydrogens=1]$1:c[allHydrogens=1]:c(-N2[allHydrogens=2]):c(-C5(=O)-O4[charge=-1]):c[allHydrogens=1]:c[allHydrogens=1]$:1 ++ C-N3[allHydrogens=2 & charge=0] >> Quinazolinone (Ladung 2) >> N2=C-N3-C5

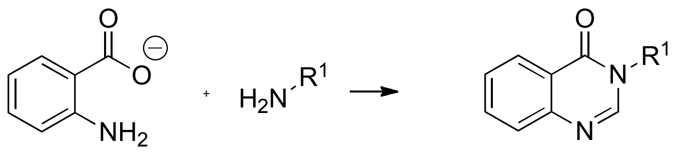

c1ccc(N)c(C(=O)[O-])c1 + CN

c[allHydrogens=1]$1:c[allHydrogens=1]:c(-N2[allHydrogens=2]):c(-C5(=O)-O4[allHydrogens=1]):c[allHydrogens=1]:c[allHydrogens=1]$:1 ++ C-N3[allHydrogens=3 & charge=1] >> Quinazolinone (Ladung 3) >> N2=C-N3-C5

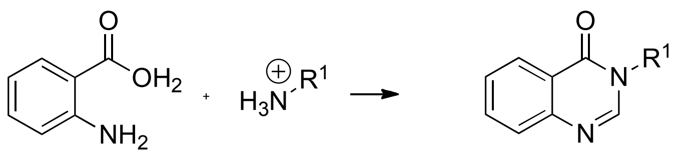

c1ccc(N)c(C(=O)O)c1 + C[NH3+]

c[allHydrogens=1]$1:c[allHydrogens=1]:c(-N2[allHydrogens=2]):c(-C5(=O)-O4[charge=-1]):c[allHydrogens=1]:c[allHydrogens=1]$:1 ++ C-N3[allHydrogens=3 & charge=1] >> Quinazolinone (Ladung 4) >> N2=C-N3-C5

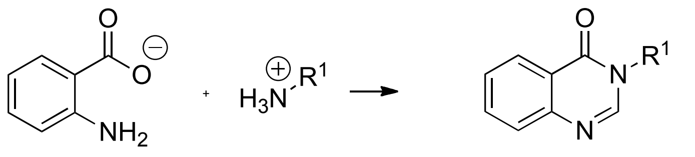

c1ccc(N)c(C(=O)[O-])c1 + C[NH3+]

12. c$1:c:c(-N-C(=O)-C1[allHydrogens=2 & !ring]-C2[!ring](=O3)-C):c4:c:c$:1 >> Chinolin-2-one intramol. >> C1=C2-c4

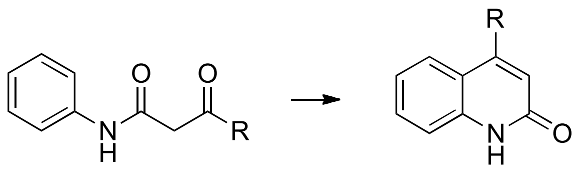

c1cc(NC(=O)CC(=O)C)ccc1

13. C-C1#N2 >> Tetrazol >> C1$1=N2-N[charge=-1]-N=N$-1

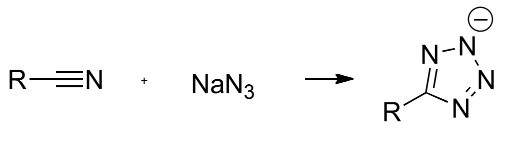

CC#N

14. C-N1[allHydrogens=2 & charge=0] ++ C-C2[allHydrogens=1](-C3(=O4)-C)-O5[allHydrogens=1] >> Tetrahydro-Indole (charge 1) >> C$1-C-C$2-N1-C3=C2-C$=2-C-C$-1

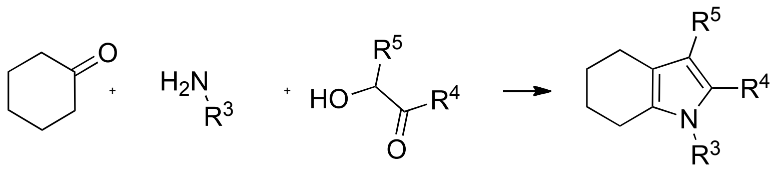

CN + CC(=O)C(O)C

C-N1[allHydrogens=3 & charge=1] ++ C-C2[allHydrogens=1](-C3(=O4)-C)-O5[allHydrogens=1] >> Tetrahydro-Indole (charge 2) >> C$1-C-C$2-N1-C3=C2-C$=2-C-C$-1

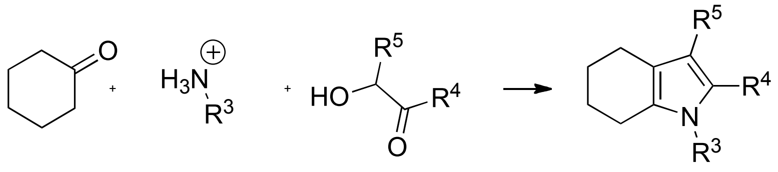

C[NH3+] + CC(=O)C(O)C

15. C1-C2[!ring](=O10)-C3[allHydrogens=2]-C4(=O11)-C5 >> 3-nitrile pyridine (symmetry 1) >> N$1=C(-O)-C(-C#N)=C2(-C1)-C3=C4$-1(-C5)

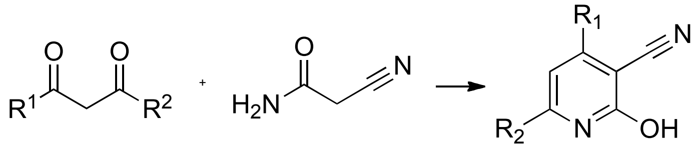

CC(=O)CC(=O)C

C1-C2[!ring](=O10)-C3[allHydrogens=2]-C4(=O11)-C5 >> 3-nitrile pyridine (symmetry 2) >> N$1=C(-O)-C(-C#N)=C2(-C5)-C3=C4$-1(-C1) CC(=O)CC(=O)C

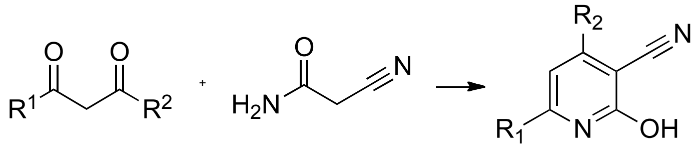

CC(=O)CC(=O)C

16. c-C1#N2[allHydrogens=0] ++ N3[allHydrogens=2]-N6[allHydrogens=1]-C4(=O5)-c >> Triazole >> C1$8=N3-N6-C4=N2$-8

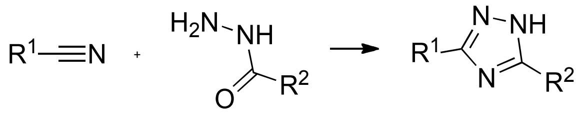

c1ccccc1C#N + NNC(=O)c1ccccc1

17. C1[sp3]-*2[Cl|Br|I] ++ C3[allHydrogens=1]#C4-C >> Huisgen 1-3 dipolar (azid in_situ) >> C1-N$1-N=N-C4=C3[bound(-H)]$-1

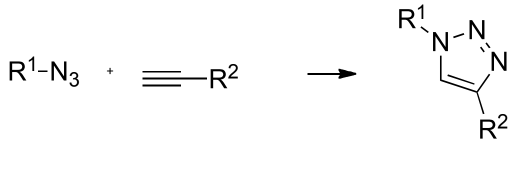

CCl + CC#C

18. C1[!aromatic]=C2[!aromatic]-C3[!aromatic]=C4[!aromatic] ++ C5[!aromatic]=C6[!aromatic] >> Diels-Alder (symmetry 1) >> C1$1-C2=C3-C4-C5-C6$-1

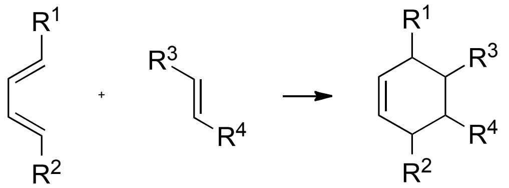

C=CC=C + C=C

C1[!aromatic]=C2[!aromatic]-C3[!aromatic]=C4[!aromatic] ++ C5[!aromatic]=C6[!aromatic]>> Diels-Alder (Symmetrie 2) >> C1$1-C2=C3-C4-C6-C5$-1

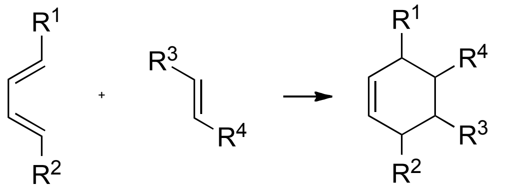

C=CC=C + C=C

19. C1[!aromatic]=C2[!aromatic & !ring]-C3[!aromatic]=C4[!aromatic] ++ C5#C6 >> Diels-Alder Alkine (symmetry 1) >> C1[!aromatic&!sp2]$1-C2[!aromatic]=C3[!aromatic]-C4[!aromatic&!sp2]-C5=C6$-1

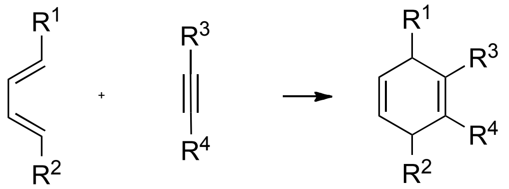

C=CC=C + C#C

C1[!aromatic]=C2[!aromatic]-C3[!aromatic]=C4[!aromatic] ++ C5#C6 >> Diels-Alder Alkine (symmetry 2) >> C1[!aromatic&!sp2]$1-C2[!aromatic]=C3[!aromatic]-C4[!aromatic&!sp2]-C6=C5$-1

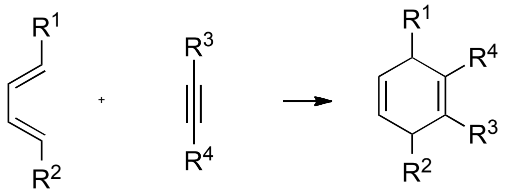

C=CC=C + C#C

20. c(-O1[allHydrogens=1]):c(-C(=O)-C2[allHydrogens=3]) ++ C[sp3]$1-C3(=O4)-C[sp3]-C[sp3]-N-C[sp3]$-1 >> Spiro-piperidine >> O1-C3-C2

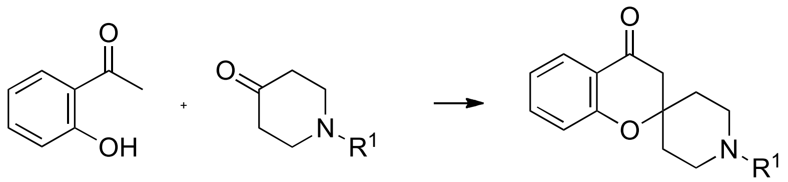

c1cc(C(=O)C)c(O)cc1 + C1C(=O)CCNC1

21. C-C1[!ring](=O6)-C2-C3(=O7)-C ++ C-N4[allHydrogens=1]-N5[allHydrogens=2] >> Pyrazole (symmetry 1) >> C1$1-N4-N5=C3-C2$=1

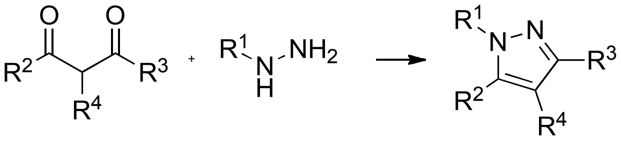


CC(=O)CC(=O)C + NNC

C-C1[!ring](=O6)-C2-C3(=O7)-C ++ C-N4[allHydrogens=1]-N5[allHydrogens=2] >> Pyrazol (symmetry 2) >> C1$1-N5-N4=C3-C2$=1

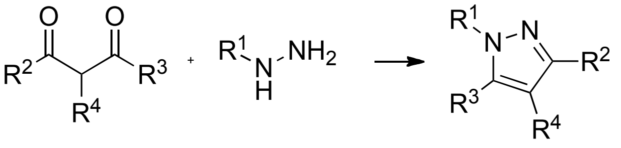


CC(=O)CC(=O)C + NNC

22. C-C1(=O5)-c:c-C2(=O6)-O7[allHydrogens=1] ++ C[!bound(=O) & !bound (=S)]-N3[allHydrogens=1]-N4[allHydrogens=2] >> Phthalazinone (charge 1) >> C2-N3-N4=C1

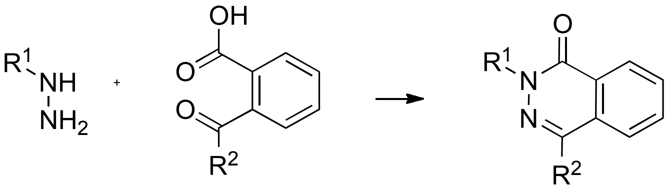


c1c(C(=O)O)c(C(=O)C)ccc1 + NNC

C-C1(=O5)-c:c-C2(=O6)-O7[charge=-1] ++ C[!bound(=O) & !bound (=S)]-N3[allHydrogens=1]-N4[allHydrogens=2] >> Phthalazinone (charge 2) >> C2-N3-N4=C1

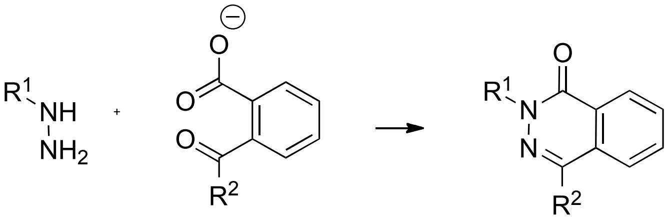


c1c(C(=O)[O-])c(C(=O)C)ccc1 + NNC

23. C-C1[!ring](=O7)-C4[!aromatic & bound(-H)]-C5[!aromatic & bound(-H)]-C2(=O6)-C ++ C[!bound(=O)]-N3[allHydrogens=2 & charge=0] >> Paal-Knorr pyrrole (charge 1) >> C1$1-N3-C2=C5-C4$=1

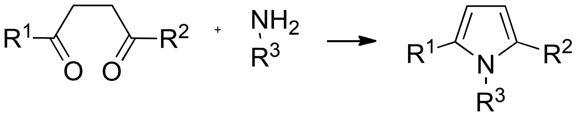


CC(=O)CCC(=O)C + CN

C-C1[!ring](=O7)-C4[!aromatic & bound(-H)]-C5[!aromatic & bound(-H)]-C2(=O6)-C ++ C[!bound(=O)]-N3[charge=1 & allHydrogens=3] >> Paal-Knorr pyrrole (charge 2) >> C1$1-N3-C2=C5-C4$=1

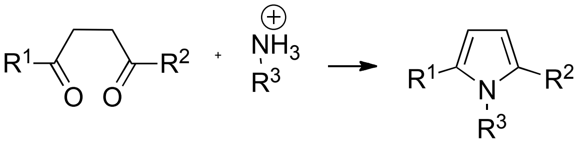


CC(=O)CCC(=O)C + C[NH3+]

24. c-C1(=O4)-C2(=O5)-c ++ C3[allHydrogens=1](=O6)-c$1:c:c:c:c:c$:1 >> Triaryl-imidazol (1,2 diketone) >> C1$1-N-C3=N-C2$=1

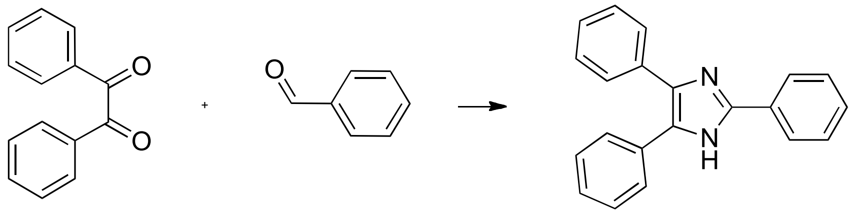

c1ccccc1C(=O)C(=O)c1ccccc1 + c1ccc(C(=O))cc1

25. c-C1(=O4)-C2(-O5[allHydrogens=1])-c ++ C3[allHydrogens=1](=O6)-c$1:c:c:c:c:c$:1 >> Triarylimidazol (alpha hydroxy-ketone) >> C1$1-N-C3=N-C2$=1

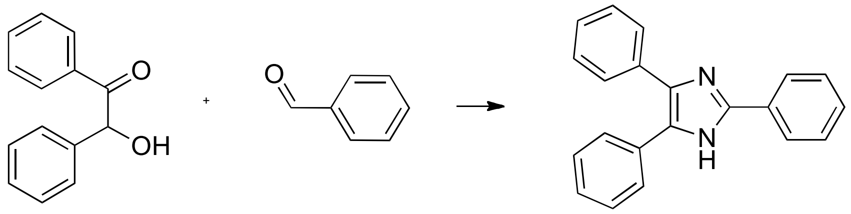

c1ccccc1C(O)C(=O)c1ccccc1 + c1ccc(C(=O))cc1

26. c$1:c4[allHydrogens=1]:c(-N5[allHydrogens=1]-N6[allHydrogens=2]):c:c:c$:1 ++ C-C1(=O2)-C3[allHydrogens=2]-C >> Fischer indole >> N5-C1=C3-c4

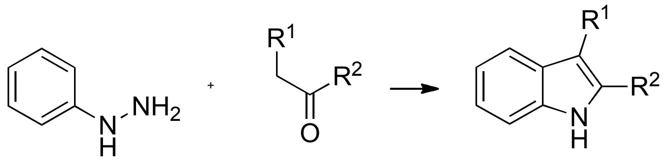

c1ccc(NN)cc1 + CC(=O)CC

27. c$1:c(-C4[allHydrogens=1](=O7)):c(-N5[allHydrogens=2]):c:c:c$:1 ++ C-C1(=O2)-C3[allHydrogens=2]-C >> Friedlaender chinoline >> N5=C1-C3=C4

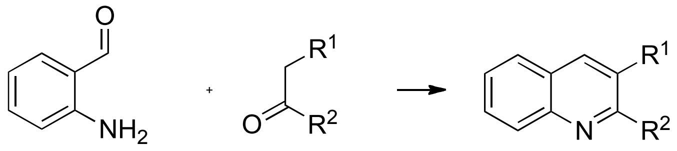

c1cc(C=O)c(N)cc1 + CC(=O)CC

28. c$1:c1[allHydrogens=1]:c(-O2[allHydrogens=1]):c:c:c$:1 ++ C-C3(=O4)-C5[allHydrogens=2 & !ring]-C6(=O)-O7-C[allHydrogens=2]-C[allHydrogens=3] >> Pechmann coumarine >> c1-C3=C5-C6-O2

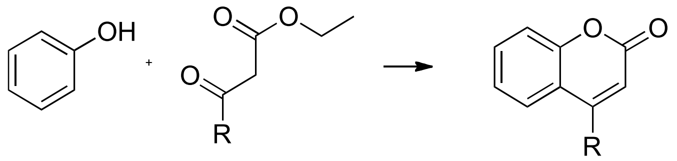

c1cc(O)ccc1 + CC(=O)CC(=O)OCC

29. c$1:c(-O1[allHydrogens=1]):c2(-I5):c:c:c$:1 ++ C3[allHydrogens=1]#C4-C >> Benzofuran >> O1-C4=C3-c2

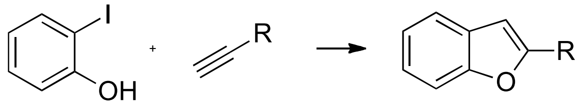


c1cc(O)c(I)cc1 + CC#C

30. C-C1(=O2)-C3[bound(-H)](-Br4) >> Imidazol-Acetamid >> C1$1=C3-N-C(-N-C(=O)-C)=N$-1

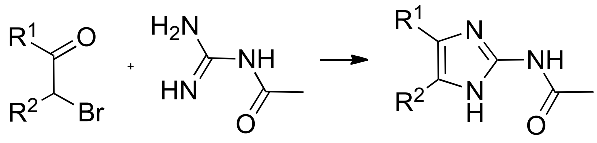


CC(=O)C(Br)

31. C[allHydrogens=3]-C[allHydrogens=2]-O1-C2[!ring](=O)-C[!aromatic]-C[!aromatic]-C[!aromatic]-C3[bound(-H) & !aromatic]-C[!ring](=O)-O-C[allHydrogens=2]-C[allHydrogens=3] >> Dieckmann 5-ring (symmetry 1) >> C2[ring]-C3

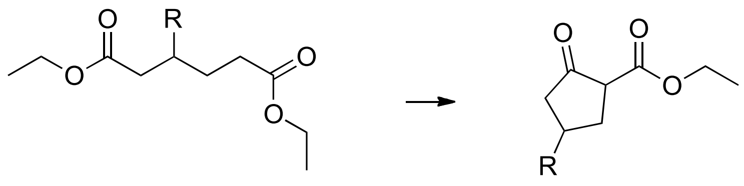

CCOC(=O)CCCCC(=O)OCC

C[allHydrogens=3]-C[allHydrogens=2]-O-C[!ring](=O)-C2[bound(-H) & !aromatic]-C[!aromatic]-C[!aromatic]-C[!aromatic]-C3[!ring](=O)-O1-C[allHydrogens=2]-C[allHydrogens=3] >> Dieckmann 5-ring (symmetry 2) >> C2-C3[ring]

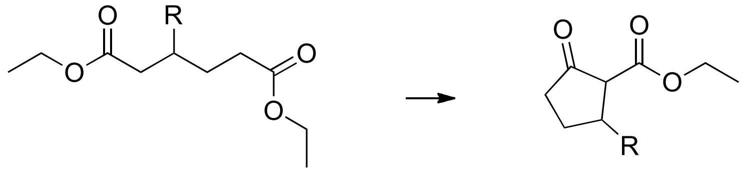

CCOC(=O)CCCCC(=O)OCC

32. C[allHydrogens=3]-C[allHydrogens=2]-O1-C2[!ring](=O)-C[!aromatic]-C[!aromatic]-C[!aromatic]-C[!aromatic]-C3[bound(-H) & !aromatic]-C[!ring](=O)-O-C[allHydrogens=2]-C[allHydrogens=3] >> Dieckmann 6-Ring (symmetry 1) >> C2-C3

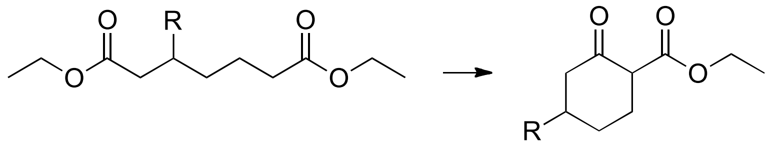

CCOC(=O)CCCCCC(=O)OCC

C[allHydrogens=3]-C[allHydrogens=2]-O-C[!ring](=O)-C2[bound(-H) & !aromatic]-C[!aromatic]-C[!aromatic]-C[!aromatic]-C[!aromatic]-C3[!ring](=O)-O1-C[allHydrogens=2]-C[allHydrogens=3] >> Dieckmann 6-Ring (symmetry 2) >> C2-C3

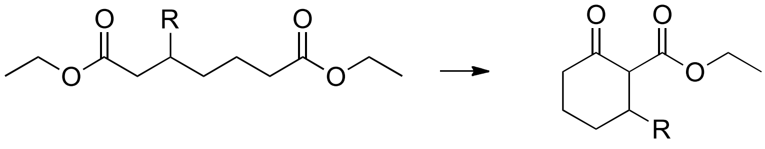

CCOC(=O)CCCCCC(=O)OCC

33. c$1:c:c(-O1[allHydrogens=1]):c(-C(=O)-C2[allHydrogens=3]):c:c$:1 ++ c$1:c:c:c(-C3(=O4)-Cl5):c[bound(-H)]:c$:1 >> Flavone >> C2=C3-O1

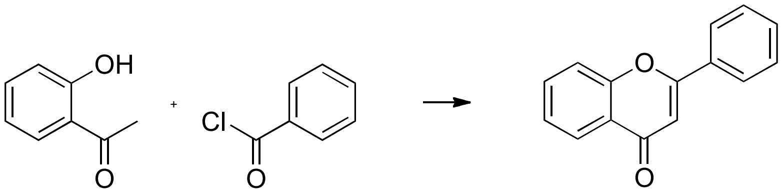

c1cc(O)c(C(=O)C)cc1 + c1ccc(C(=O)Cl)cc1

34. c-C1#N2 ++ C3[allHydrogens=0](=O4)-O5[allHydrogens=1] >> Oxadiazole (charge 1) >> C1$1=N-O-C3=N2$-1

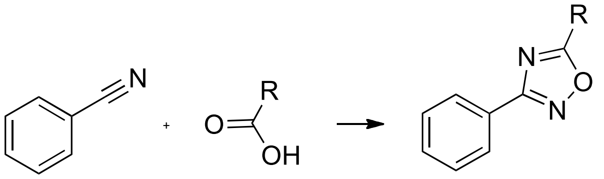

c1cc(C#N)ccc1 + CC(=O)O

c-C1#N2 ++ C3[allHydrogens=0](=O4)-O5[charge=-1] >> Oxadiazole (charge 2) >> C1$1=N-O-C3=N2$-1

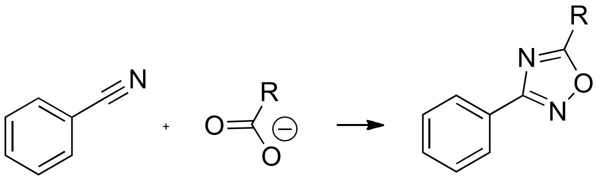

c1cc(C#N)ccc1 + CC(=O)[O-]

35. C(=O)(-*[{O & allHydrogens=0} | C])-C1[allHydrogens=2]-C(=O)-*[{O & allHydrogens=0} | C] ++ C2[!aromatic]=C3[!aromatic]-C4(=O)-C >> Michael addition >> C1-C2-C3-C4

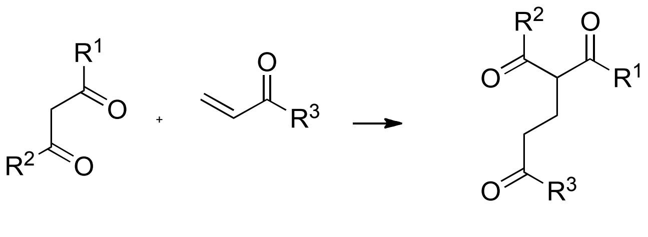

CC(=O)CC(=O)C + C=CC(=O)C

36. *[{C & !bound(-H)}|{O & !bound(-H) & charge=0}]-C1[!ring](=O)-O2-C[!ring] ++ C3[allHydrogens=2]-C(=O)-*[{C & !bound(-H)}|{O & !bound(-H) & charge=0}] >> crossed Claissen >> C3-C1

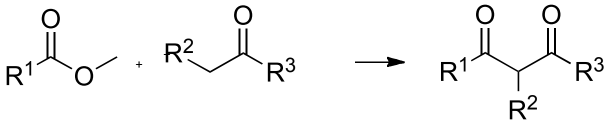

c1ccccc1C(=O)OC + CCC(=O)OC

37. c-O1[allHydrogens=1] ++ C2[allHydrogens=2]-*3[I|Br|Cl] >> Williamson ether >> O1-C2

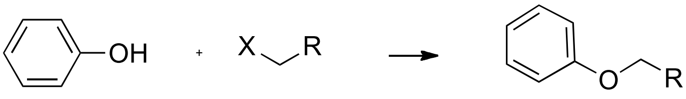


c1cc(O)ccc1 + CCBr

38. C-C1(=O2)-C ++ N3[allHydrogens=2 &charge=0]-C[!bound(=O) & !bound(=N)] >> red. amination (one step), ketone, prim. amine (charge 1) >> C1-N3[charge=1]

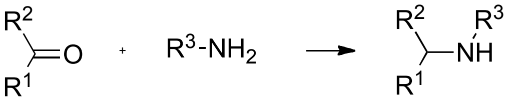

CC(=O)C + CN

C-C1(=O2)-C ++ N3[allHydrogens=3 &charge=1]-C[!bound(=O) & !bound(=N)] >> red. amination ketone, prim. amine (charge 2) >> C1-N3[charge=1]

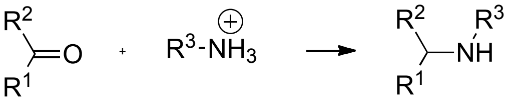

CC(=O)C + C[NH3+]

39. C-C1[bound(-H)](=O2) ++ N3[allHydrogens=2 &charge=0]-C[!bound(=O) & !bound(=N)] >> red. amination, aldehyde, prim. amine (charge 1) >> C1-N3[charge=1]

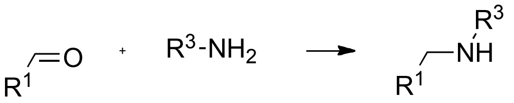

CC(=O) + CN

C-C1[bound(-H)](=O2) ++ N3[allHydrogens=3 &charge=1]-C[!bound(=O) & !bound(=N)] >> red. amination, aldehyde, prim. amine (charge 2)>> C1-N3[charge=1]

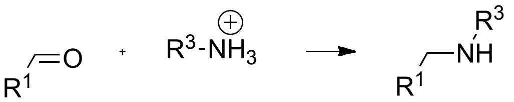

CC(=O) + C[NH3+]

40. C-C1(=O2)-C ++ C[!bound(=O) & !bound(=N)]-N3[allHydrogens=1 & charge=0 & !aromatic]-C[!bound(=O) & !bound(=N)] >> red. amination, ketone, sec. amine (charge 1) >> C1-N3[charge=1]

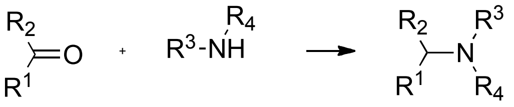

CC(=O)C + CNC

C-C1(=O2)-C ++ C[!bound(=O) & !bound(=N)]-N3[allHydrogens=2 & charge=1 & !aromatic]-C[!bound(=O) & !bound(=N)] >> red. amination, ketone, sec. amine (charge 2) >> C1-N3[charge=1]

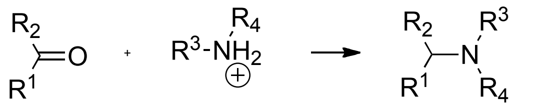

CC(=O)C + C[NH2+]C

41. C-C1[bound(-H)](=O2) ++ C[!bound(=O) & !bound(=N)]-N3[allHydrogens=1 & charge=0 & !aromatic]-C[!bound(=O) & !bound(=N)] >> red. amination, aldehyde, sec. amine (charge 1) >> C1-N3[charge=1]

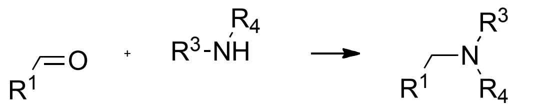

CC(=O) + CNC

C-C1[bound(-H)](=O2) ++ C[!bound(=O) & !bound(=N)]-N3[allHydrogens=2 & charge=1 & !aromatic]-C[!bound(=O) & !bound(=N)] >> red. amination, aldehyde, sec. amine (charge 2) >> C1-N3[charge=1]

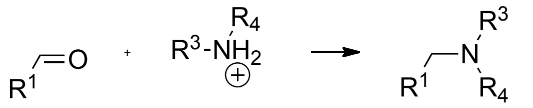

CC(=O) + C[NH2+]C

42. C1[sp2]-B3(-O)-O ++ C2[sp2 & !bound(=O)]-*4[Cl|Br|I]>> Suzuki >> C1-C2

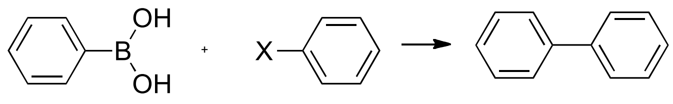

c1cc(B(O)(O))ccc1 + c1cc(Cl)ccc1

43. c[allHydrogens=1]$1:c:c:c[allHydrogens=1]:c$2-N[allHydrogens=1]-C=C5[allHydrogens=1]-c$:1$:2 ++ C[allHydrogens=2]$3-N-C[allHydrogens=2]-C[allHydrogens=2]-C4(=O7)-C6[allHydrogens=2]$-3 >> Piperidine+Indole >> C4(=C6)-C5

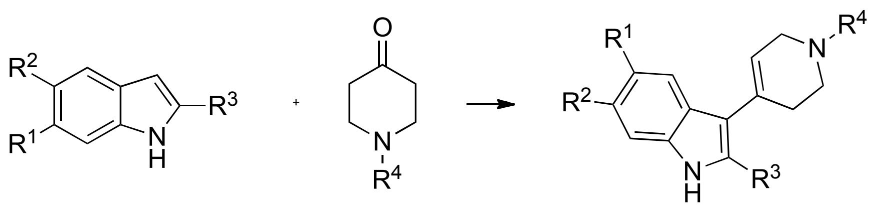

c1cc2NC=Cc2cc1 + C1C(=O)CCNC1

44. C1[!bound(=O)]-*2[Br|Cl|I] ++ *5[Br|Cl]-C4[allHydrogens=2]-C[allHydrogens=2] >> Negishi >> C1-C4

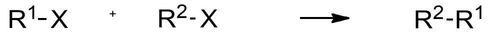

CI + CCBr

45. C1[bound(-H) & !bound(=O)]-O2[allHydrogens=1] ++ C(=O)-N3[allHydrogens=1]-C(=O) >> Mitsunobu (imide) >> C1-N3

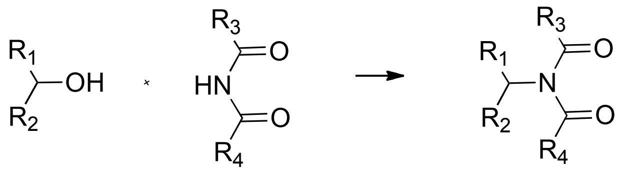

CC(O)C + C(=O)NC(=O)

46. C1[bound(-H) & !bound(=O)]-O2[allHydrogens=1] ++ C-C(=O)-O3[allHydrogens=1] >> Mitsunobu Carbonsäure (carbon acid, charge 1) >> C1-O3

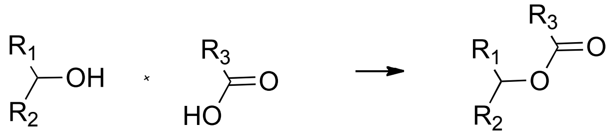

CC(O)C + CC(=O)O

C1[bound(-H) & !bound(=O)]-O2[allHydrogens=1] ++ C-C(=O)-O3[charge=-1] >> Mitsunobu (carbon acid, charge 2) >> C1-O3 CC(O)C CC(=O)[O-]

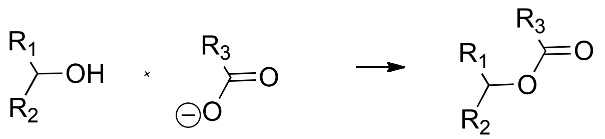

47. C1[bound(-H) & !bound(=O)]-O2[allHydrogens=1] ++ C-N3[bound(-H)]-S(=O)(=O)-C >> Mitsunobu Sulfonic amide >> C1-N3

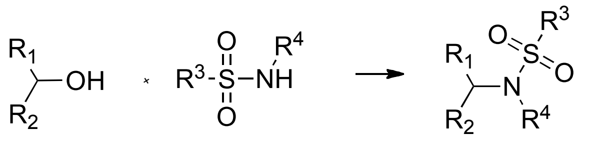

CC(O)C + CNS(=O)(=O)C

48. C1[!bound(=O)]-*3[Br | I | Cl] ++ C-C2[allHydrogens=1 & !aromatic]=C[!aromatic](-C)-C >> Heck >> C1-C2

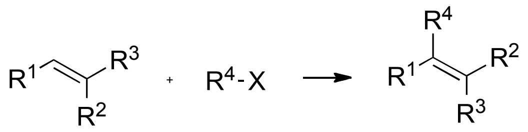

CBr + CC(=CC)C

49. C-C1(=O)-Cl2 ++ C[!bound(=O) & !bound(=N)]-N3[allHydrogens=2 & charge=0] >> Amide, prim. amine (charge 1) >> C1-N3

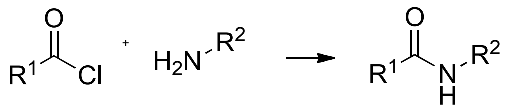

CC(=O)Cl + CN

C-C1(=O)-Cl2 ++ C[!bound(=O) & !bound(=N)]-N3[allHydrogens=3 & charge=1] >> Amide, prim. amine (charge 2) >> C1-N3

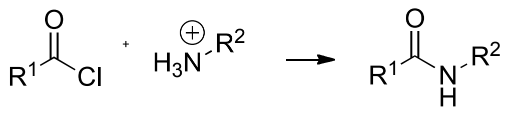

CC(=O)Cl + C[NH3+]

50. C-C1(=O)-Cl2 ++ C[!bound(=O) & !bound(=N)]-N3[allHydrogens=1 & charge=0]-C[!bound(=O) & !bound(=N)] >> Amide, sec. amine (charge 1) >> C1-N3

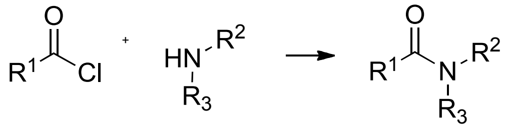

CC(=O)Cl + CNC

C-C1(=O)-Cl2 ++ C[!bound(=O) & !bound(=N)]-N3[allHydrogens=2 & charge=1]-C[!bound(=O) & !bound(=N)] >> Amide, sec. amine (charge 2) >> C1-N3

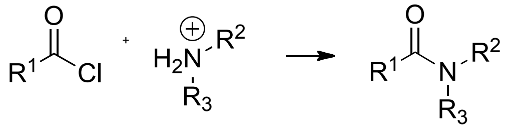

CC(=O)Cl + C[NH2+]C

51. C-C1(=O)-Cl2 ++ C[!bound(=O)]-O3[allHydrogens=1] >> Ester >> C1-O3

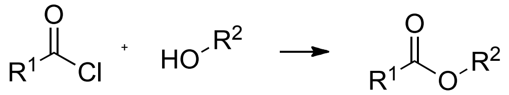

CC(=O)Cl + CO

52. c-C1[!aromatic]=C2[!aromatic & allHydrogens=2] ++ S3[allHydrogens=1]-C >> Thioether >> C1-C2-S3

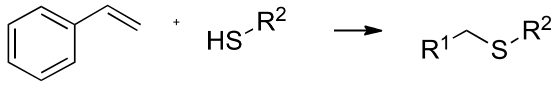

c1ccccc1C=C + CS

53. C-C1(=O)-Cl2 ++ C3-I4 >> Ketone >> C1-C3

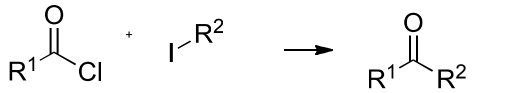

CC(=O)Cl + CI

54. C-S1(=O)(=O)-Cl3 ++ N2[allHydrogens=2 & charge=0]-C[!bound(=O) & !bound(=N)] >> Sulfonamid (Ladung 1) >> S1-N2

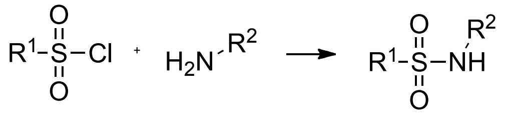

CS(=O)(=O)Cl + CN

C-S1(=O)(=O)-Cl3 ++ N2[allHydrogens=3 & charge=1]-C[!bound(=O) & !bound(=N)] >> Sulfonamid (Ladung 2) >> S1-N2

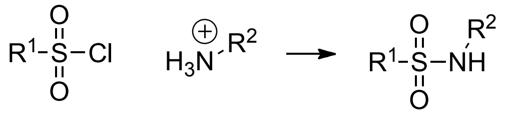

CS(=O)(=O)Cl + C[NH3+]

55. c1-B2(-O[allHydrogens=1])(-O[allHydrogens=1]) ++ c$1:n3[allHydrogens=1]:n:c:c$:1 >> Ar-Pyrazole >> c1-N3

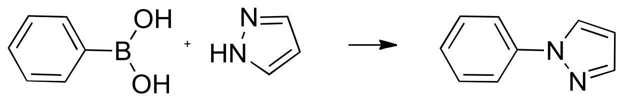

c1cc(B(O)(O))ccc1 + C1=CC=NN1

56. c1-B2(-O[allHydrogens=1])(-O[allHydrogens=1]) ++ c$1:n3[allHydrogens=1]:c:n:c$:1 >> Ar-Imidazole >> c1-N3

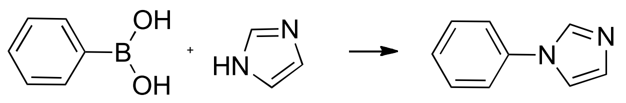

c1cc(B(O)(O))ccc1 + C1=CN=CN1

57. C1[sp3]-*2[Cl|Br|I] ++ C3[allHydrogens=1]#C >> Alkine alkylation >> C1-C3

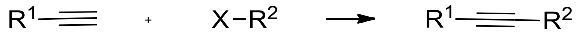

CCl + C#C

58. C-C2(=O)-Cl4 ++ C3[allHydrogens=1]#C >> Alkine acylation >> C2-C3

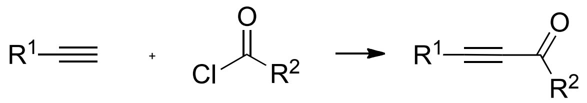

CC(=O)Cl + C#C

**Table S2.** Preprocessing reactions. Each reaction is specified by (i) Reaction-MQL expression, (ii) schematic structural representation.

1. C1(=O2)-O3[allHydrogens=1] >> FGI Acyl chloride (charge 1) >> C1(=O2)-Cl

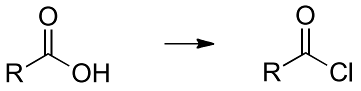


C1(=O2)-O3[charge=-1] >> FGI acyl Chloride (charge 2) >> C1(=O2)-Cl

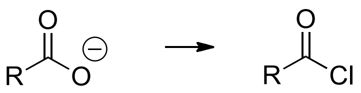


2. C1[aliphatic & !bound(=O) & !bound(=S)]-O2[allHydrogens=1] >> FGI bromination >> C1-Br

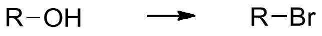


3. C1[aliphatic & !bound(=O) & !bound(=S)]-O2[allHydrogens=1] >> FGI chlorination >> C1-Cl

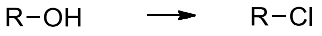


4. C-S1(=O)(=O)-O2[allHydrogens=1] >> FGI sulfonyl chloride (charge 1)>> S1-Cl

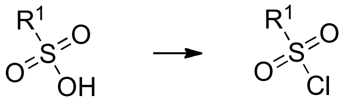


C-S1(=O)(=O)-O2[charge=-1] >> FGI sulfonyl chloride (charge 2) >> S1-Cl

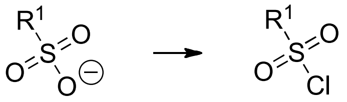


5. C1[!aromatic & allHydrogens=2 & !bound(-Halogen)]-C(=O)-O[allHydrogens=1] >> FGA alpha bromination (charge 1) >> C1-Br

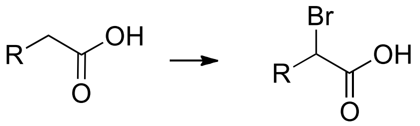


C1[!aromatic & allHydrogens=2 & !bound(-Halogen)]-C(=O)-O[charge=-1] >> FGA alpha bromination (charge 2) >> C1-Br

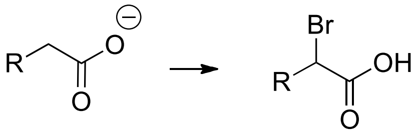


6. C1[!aromatic & allHydrogens=2 & !bound(-Halogen)]-C(=O)-O[allHydrogens=1] >> FGA alpha chlorination (Ladung 1) >> C1-Cl

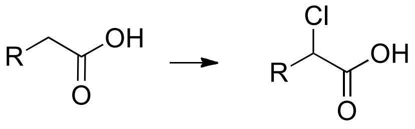


C1[!aromatic & allHydrogens=2 & !bound(-Halogen)]-C(=O)-O[charge=-1] >> FGA alpha chlorination (charge 2) >> C1-Cl

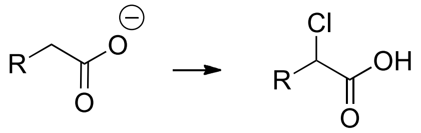

7. c1-*2[Cl|Br] >> FGI Rosenmund-von-Braun >> c1-C#N

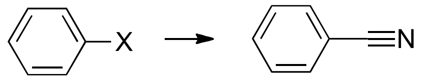

8. C-C1[allHydrogens=2]-O2[allHydrogens=1] >> FGI nitrilation prim. hydroxy >> C1#N

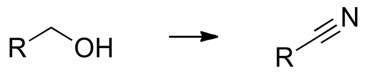

9. C-C1[allHydrogens=2]-N2[allHydrogens=2 & charge=0] >> FGI nitrilation prim. amine (charge 1) >> C1#N2

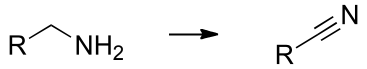

C-C1[allHydrogens=2]-N2[allHydrogens=3 & charge=1] >> FGI nitrilation prim. Aminen (charge 2) >> C1#N2

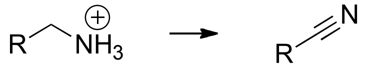

10. C-C1#C2[allHydrogens=1] >> FGI nitrilation term. alkine >> C1#N

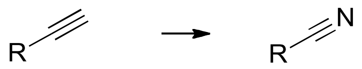

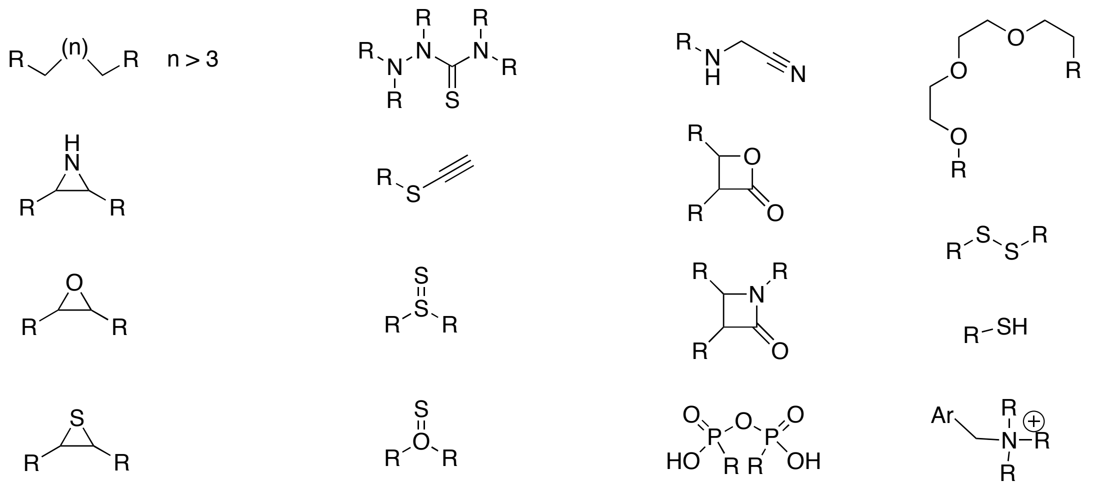


**Figure S1.** Unwanted substructures. The set of unwanted substructures is based on definitions described by Hann et al. [1]. Building blocks containing one of these substructures are removed from the stock of building blocks (Ar: aromatic).

**Table S3.** Pharmacophore typing. The order of rules represents the order of there execution and does matter: once an atom has been typed it cannot be typed by any subsequent rule.

| MQL substructure definition | Type | Example |
|---|---|---|
| O[charge=-1]-N[charge=1]=O | 0;0;0 | 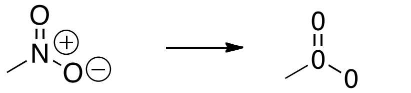 |
| *[charge>0] | P | 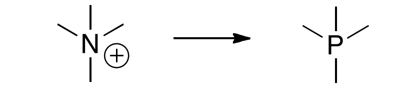 |
| *[charge<0] | N | 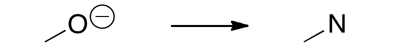 |
| O=C-N[allHydrogens>0] | A;0;D | 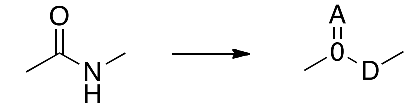 |
| O=C-N[allHydrogens=0] | A;0;0 | 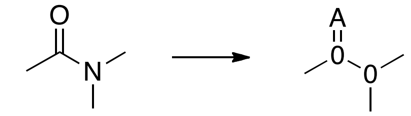 |
| O=C-O[allHydrogens=0] | A;0;0 | 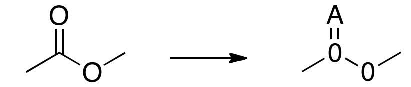 |
| N[allHydrogens>0 & !aromatic & !bound(-C=N) & !bound(-S=O)] | E | 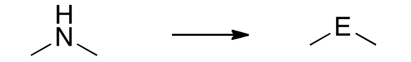 |
| O-H' | E | 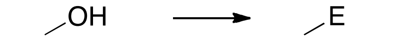 |
| Heavy'[!aromatic]-O-Heavy'[!aromatic] | A | 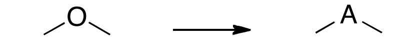 |
| N[allHydrogens=0 & !{aromatic&totalConnections=3} & !{bound(-C=N) & !bound(=C)} & !{bound(-C:N) & !bound(=C)} & !bound(-S=O)] | A | 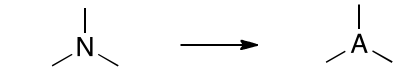 |
| O=*'[C\|P\|S\|N] | A | 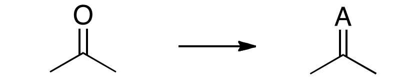 |
| N[{allHydrogens=1 & aromatic} \| {allHydrogens>0 & bound(-C=N)} \| {allHydrogens>0 & bound(-S=O)}] | D | 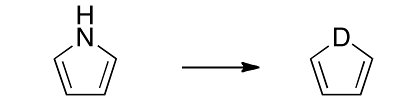 |
| N[aromatic] | R | 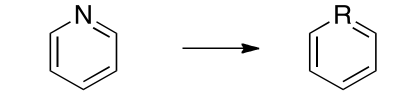 |
| O[aromatic] | R | 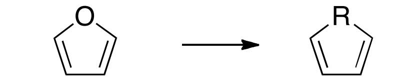 |
| C[aromatic] | R | 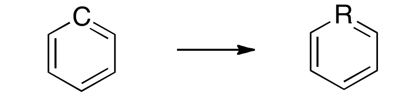 |
| S[aromatic] | R | 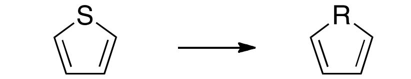 |
| Cl | L | 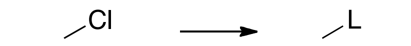 |
| Br | L | 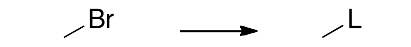 |
| I | L | 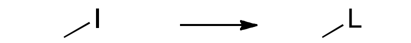 |
| C[!bound(~N)&!bound(~O)]~*'[C\|F\|Cl\|Br\|I\|S] | L | 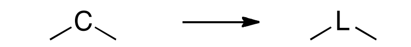 |
| S[!bound(~N)&!bound(~O)]~*'[C\|H] | L | 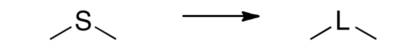 |

**Table S4.** Synthetic accessibility scores. Average *rsynth* descriptor values of compounds designed by DOGS (values in parentheses represent the standard error of mean).

| reference | rsynth | rsynth (av.) mol. graph | rsynth (av.) red. graph |
|---|---|---|---|
| 1 | 1.0 | 0.98 (±0.006) | 0.95 (±0.009) |
| 2 | 1.0 | 0.98 (±0.007) | 0.91 (±0.012) |
| 3 | 1.0 | 0.96 (±0.008) | 0.91 (±0.013) |
| 4 | 1.0 | 0.85 (±0.015) | 0.83 (±0.027) |
| 5 | 0.37 | 0.79 (±0.014) | 0.76 (±0.035) |

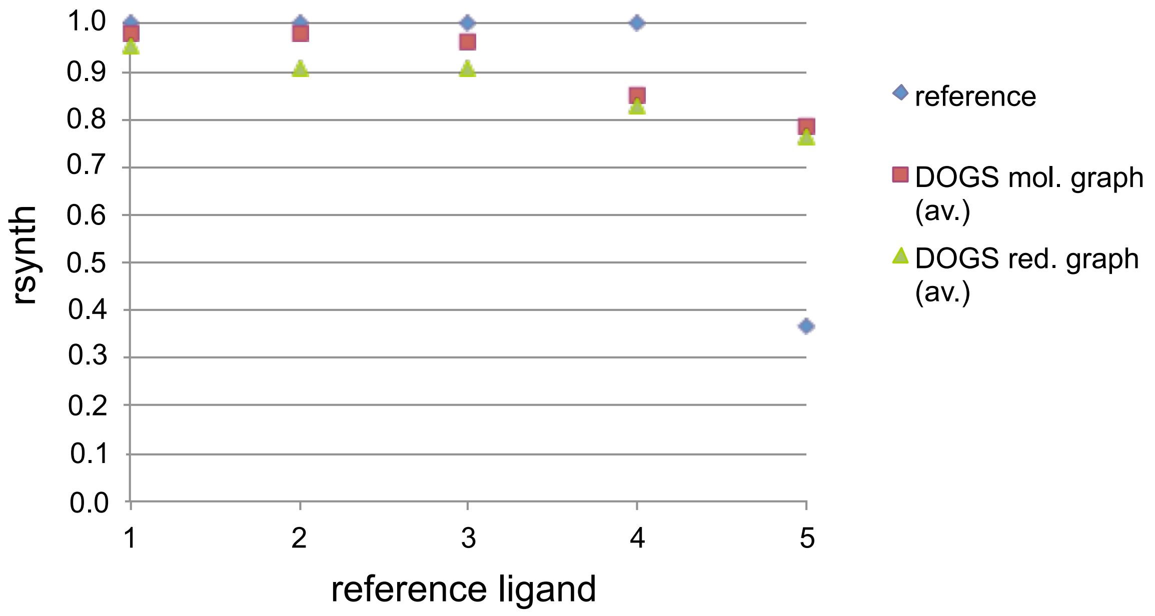


**Figure S2.** Synthetic accessibility scores. Synthetic accessibility scores (*rsynth*) of five trypsin reference compounds and corresponding DOGS molecules (averaged over all molecules of a run) based on the molecular graph representation and reduced graph representation.

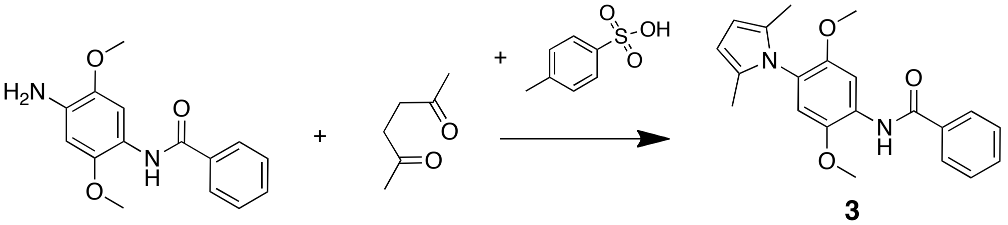


**3**

**Figure S3.** Synthesis of compound **3** as proposed by the software.

**Protocol S1.** Preparation of compound **3**

**Compound 3 (*N*-(4-(2,5-dimethyl-1*H*-pyrrol-1-yl)-2,5-dimethoxyphenyl)benzamide)**: N-(4-amino-2,5-dimethoxyphenyl)benzamide (3.0 eq), hexane-2,5-dione (1.0 eq), *p*-toluenesulfonic acid (2.0 eq), and 4Å molecular sieves (spatula tip) were dissolved in ethanol (5 ml) and heated in the microwave for 15 minutes at 160 °C [2]. Upon cooling to room temperature, the reaction mixture was diluted with ethyl acetate and washed with water. The aqueous layer was washed with ethyl acetate and the combined the organic extracts were washed with brine, dried over magnesium sulfate, filtered, and concentrated *in vacuo*. The resulting residue was purified by flash column chromatography (ethyl acetate/n-hexane) to give compound **3**. 1H-NMR (400.13MHz, DMSO-*d6*,) $\delta$ 1.99 (s, 6H), 3.73 (s, 3H), 3.88 (s, 3H), 5.82 (s, 2H), 6.99 (s, 1H), 7.89 (s, 1H), 7.59-7.70 (m, 3H), 8.03 (dd, 2H), 9.55 (s, 1H). 13C-NMR (100.61MHz, DMSO-*d6*) $\delta$ 12.46, 56.06, 56.59, 105.21, 108.73, 119.60, 133.40, 127.44, 127.92, 128.56, 131.78, 140.89, 148.75, 163.20. HRMS (ESI$^+$): *m/z* [M + H]$^+$ calculated for $C_{21}H_{23}N_2O_3$: 351.1703, measured: 351.1701. HPLC-MS (acetonitrile/$H_2O$): purity >99%.

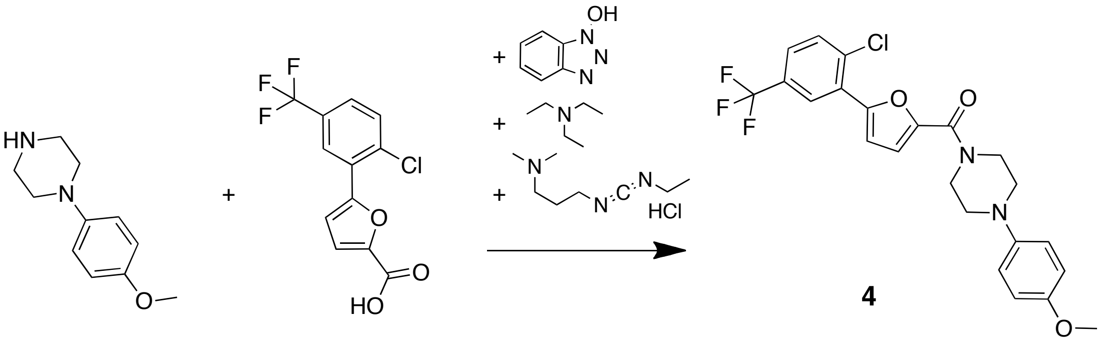


**4**

**Figure S4.** Synthesis of compound **4** as proposed by the software.

**Protocol S2.** Preparation of compound **4**

**Compound 4 ((5-(2-chloro-5-(trifluoromethyl)phenyl)furan-2-yl)(4-(4-methoxyphenyl)piperazin-1-yl)methanone)**: 1-(4-methoxyphenyl)piperazine (1.0 eq), EDCI (1.5 eq), HOBT (2.0 eq), TEA (2.0 eq) and 5-(2-chloro-5-(trifluoromethyl)phenyl)furan-2-carboxylic acid (1.2 eq) were dissolved in dry dichloromethane (10 ml) and stirred at ambient temperature overnight [3]. The mixture was concentrated on silica gel. Flash column chromatography (n-hexane/ethyl acetate) and recrystallization from hexane/ethyl acetate yielded pure compound **4** as an amorphous yellow solid. $^{1}$H-NMR (400.13MHz, DMSO-*d6*)

3.10 (t, 4H), 3.69 (s, 3H), 3.86 (br s), 6.85 (d, 2H), 6.94 (d, 2H), 7.22 (d, 1H), 7.40 (d, 1H), 7.78 (dd, 1H), 7.86 (d, 1H), 8.12 (d, 1H). $^{13}$C-NMR (100.61MHz, DMSO-*d6*) 50.07, 55.15, 113.28, 114.30, 117.18, 117.99, 122.15, 124.86, 126.16, 132.23, 145.00, 147.13, 148.91, 153.36, 157.87. HRMS (ESI$^{+}$): *m/z* $[M + H]^{+}$ calculated for $C_{23}H_{21}ClF_3N_2O_3$: 465.1187, measured: 465.1180. HPLC-MS (acetonitrile/$H_2O$): purity >99%.

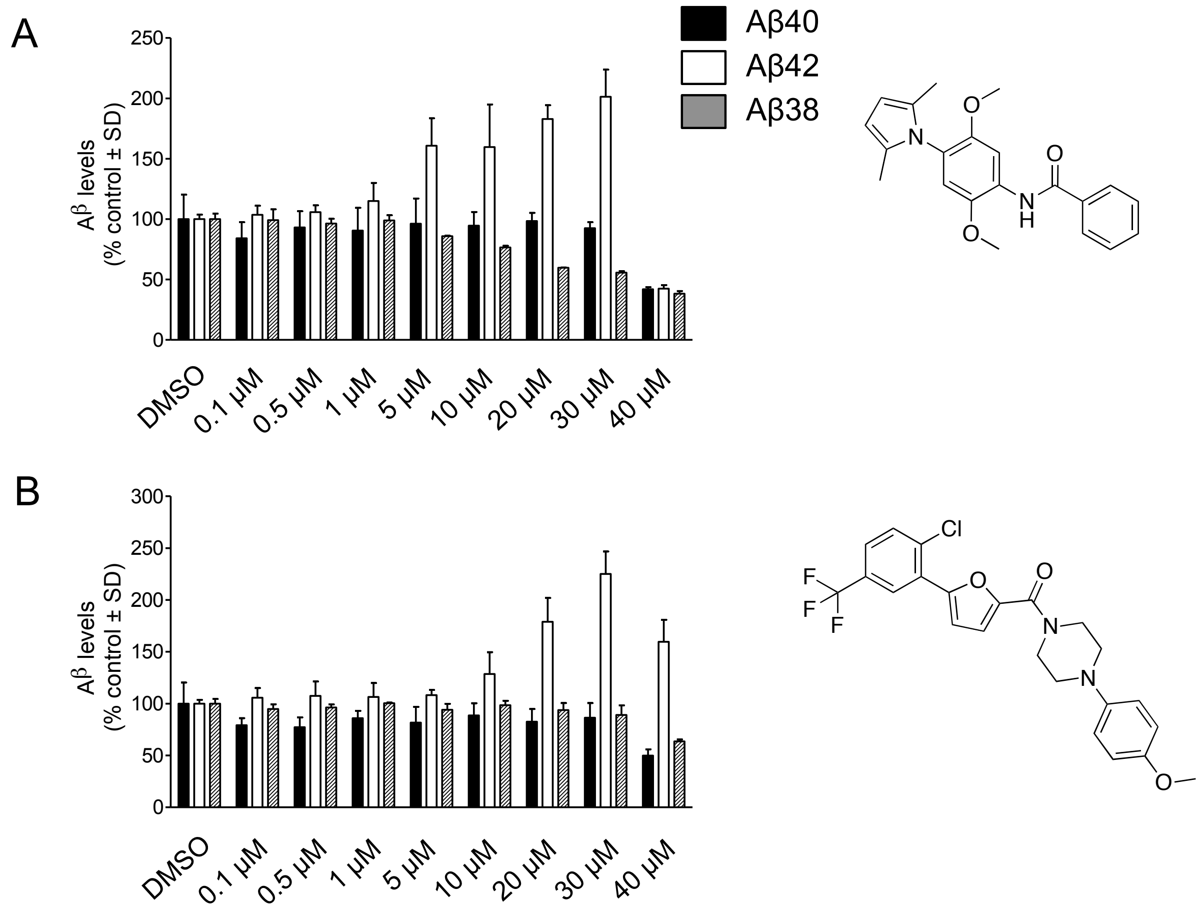


**Figure S5.** Modulation of γ-secretase activity by designed compounds **3** (A) and **4** (B). Both compounds behave as inverse modulators of γ-secretase and shift the product ratio towards higher levels of Aβ42. Concentrations of secreted Ab peptides were detected in cell supernatants by sandwich ELISA using C-terminus specific antibodies that distinguish between Aβ38, Aβ40, and Aβ42 peptide species as previously described [4].

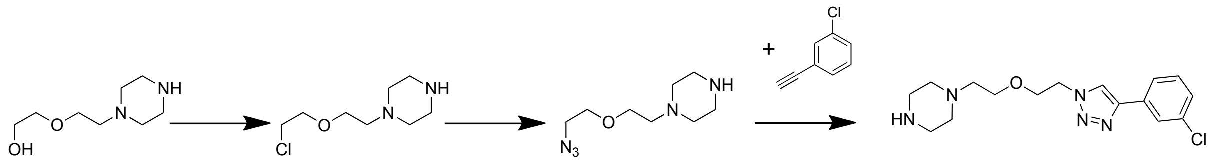

7.1 7.2 7

**Figure S6.** Synthesis of compound **7** as proposed by the software.

**Protocol S3.** Preparation of compound **7**

**Compound 7.1 (1-(2-(2-chloroethoxy)ethyl)piperazine).** 2-(2-(piperazin-1-yl)ethoxy)ethanol (1 eq) was precipitated with 5N isopropylic HCl (3 eq). The salt was filtered off and dried. In order to substitute the hydroxy group with chloride, the salt (1 eq.) was dissolved in toluene, and thionyl chloride (3 eq) was added slowly under cooling conditions (ice bath). After heating to 70°C for 10 minutes, the mixture was stirred for 3h at 60°C under argon atmosphere. The formed precipitate was filtered off and dried *in vacuo* to yield a yellowish-white solid. MS ($ESI^+$): *m/z* = 192.91 $[M+H]^+$. $^1H$ NMR (400.13 MHz, MeOD): $\delta$ 3.57 (t, 2H), 3.63 (m, 8H), 3.77 (t, 2H), 3.85 (t, 2H), 3.97 (t, 2H).

**Compound 7.2 (1-(2-(2-azidoethoxy)ethyl)piperazine).** Compound **7.1** (1 eq) and sodium azide (2 eq) were dissolved in DMSO. The mixture was stirred for 42h at 100°C. The precipitated white solid was removed by filtration. The orange filtrate was diluted with dichloromethane and extracted with 2N NaOH (three times). After removal of the solvent, the brown product (oil) was dried *in vacuo*. MS ($ESI^+$): *m/z* = 199.93 $[M + H]^+$. $^1H$ NMR (400.13 MHz, DMSO-*d6*): $\delta$ 2.34 (t, 4H), 2.44 (t, 2H), 2.69 (t, 4H), 3.38 (t, 2H), 3.54 (t, 2H), 3.58 (t, 2H).

**Compound 7 (1-(2-(2-(4-(3-chlorophenyl)-1*H*-1,2,3-triazol-1-yl)ethoxy)ethyl)piperazine).** Compound **7.2** (1 eq.) and 1-chloro-3-ethynylbenzene (1eq) were dissolved in a mixture of water and isopropyl alcohol (1:1) and placed in a 5ml microwave vial. Copper(I)-iodide (0.1 eq) was added and the mixture was heated in a microwave oven (Biotage Initiator, 100W, 125°C, 20min, absorption level: high). The mixture was extracted three times with dichloromethane and 2N NaOH. After removal of the solvent, the remaining oil was purified by flash column chromatography to yield compound **7** as a light brown oil. MS (ESI+): *m/z* = 335.82 $[M + H]^+$. $^1H$ NMR (400.13 MHz, DMSO-*d6*): $\delta$ 2.25 (t, 4H), 2.39 (t, 2H), 2.60 (t, 4H), 3.52 (t, 2H), 3.84 (t, 2H), 4.57 (t, 2H), 7.40 (ddd, 1H), 7.49 (t, 1H), 7.83 (dt, 1H), 7.9 (t, 1H), 8.65 (s, 1H). $^{13}C$ NMR (100.61 MHz, DMSO-*d6*): $\delta$ 48.22, 49.46, 54.74, 64.42, 68.57, 122.72, 123.69, 124.67, 127.58, 130.86, 132.87, 133.68, 144.93. HRMS ($ESI^+$): *m/z* $[M + H]^+$ calculated for $C_{16}H_{23}ClN_5O$: 336.1586; found: 336.1586. HPLC-MS ($MeOH/H_2O$): purity >99%.

## References


1. Hann M et al. (1999) Strategic pooling of compounds for high-throughput screening. J Chem Inf Comput Sci 39: 897-902.
2. Bharadwaj AR, Scheidt KA (2004) Catalytic multicomponent synthesis of highly substituted pyrroles utilizing a one-pot sila-Stetter/Paal-Knorr strategy. Org Lett 6: 2465-2468.

3. Brown DA, Mishra M, Zhang S, Biswas S, Parrington I, Antonio T, Reith MEA, Dutta AK (2009) Investigation of various N-heterocyclic substituted piperazine versions of 5/7-{[2-(4-aryl-piperazin-1-yl)-ethyl]-propyl-amino}-5,6,7,8-tetrahydro-naphthalen-2-ol: Effect on affinity and selectivity for dopamine D3 receptor. Bioorg Med Chem 17, 3923-3933.
4. Hieke M, Ness J, Steri R, Dittrich M, Greiner C, Werz O, Baumann K, Schubert-Zsilavecz M, Weggen S, Zettl H. (2010) Design, synthesis, and biological evaluation of a novel class of gamma-secretase modulators with PPARgamma activity. J Med Chem 53, 4691-4700.
